# Machine learning-assisted optimization of ultrasound–ohmic processing of hawthorn vinegar: antidiabetic activity, phenolic profiling, and molecular docking insights

**DOI:** 10.3389/fnut.2026.1889843

**Published:** 2026-07-08

**Authors:** Dilek Dülger Altıner, Mehmet Ali Şimşek, Mehmet Ali Yalçınkaya, Seydi Yıkmış, Elvan Üstün, Nazan Tokatlı Demirok, Melikenur Türkol, Marwa Ezz El-Din Ibrahim, Deniz Aktaran Bala

**Affiliations:** 1 Department of Gastronomy and Culinary Arts, Tourism Faculty, Kocaeli University, Kartepe, Türkiye; 2 Department of Software Engineering, Faculty of Engineering and Natural Sciences, Bandırma Onyedi Eylül University, Bandırma, Balıkesir, Türkiye; 3 Department of Computer Engineering, Faculty of Engineering and Architecture, Kırşehir Ahi Evran University, Kırşehir, Türkiye; 4 Department of Food Technology, Tekirdag Namik Kemal University, Tekirdag, Türkiye; 5 Department of Chemistry, Faculty of Art and Science, Ordu University, Ordu, Türkiye; 6 Nutrition and Dietetics, Faculty of Health Sciences, Tekirdag Namik Kemal University, Tekirdag, Türkiye; 7 Department of Food and Nutrition Sciences, College of Agricultural and Food Sciences, King Faisal University, Al-Ahsa, Saudi Arabia; 8 Department of Food Processing, Vocational School of Veterinary Medicine, Istanbul University-Cerrahpasa, Istanbul, Türkiye

**Keywords:** antidiabetic activity, hawthorn vinegar, machine learning, molecular docking, ultrasound–ohmic processing

## Abstract

Hawthorn vinegar is a fermented product with functional properties, containing phenolic compounds and bioactive ingredients that could promote health. In this study, ultrasound–ohmic (USOH) processing conditions were optimized using a hybrid machine-learning-based approach to maximize the α-glucosidase and α-amylase and inhibitory activities of hawthorn vinegar. A Box–Behnken experimental design with 27 runs was used, including four independent variables: ultrasound amplitude (40–80%), ultrasound duration (2–6 min), ohmic field strength (20–40 V/cm), and ohmic heating time (2–6 min). Thirteen machine learning algorithms were comparatively evaluated using systematic hyperparameter optimization with GridSearchCV and 5-fold cross-validation. The Lasso Poly2 model showed the highest predictive performance for both α-glucosidase and α-amylase inhibition, with CV *R*^2^ values of 0.9301 and 0.9299, respectively, and low MAPE values (<1%). Metaheuristic optimization algorithms, including Particle Swarm Optimization (PSO), Differential Evolution (DE), and Gray Wolf Optimization (GWO), converged to similar optimum processing conditions, indicating the robustness of the optimized process region. Under the combined optimal conditions, the experimental α-amylase and α-glucosidase inhibition activities were 39.27 ± 1.36% and 37.54 ± 0.53%, respectively. In addition, USOH treatment significantly enhanced the phenolic profile of hawthorn vinegar compared to thermally pasteurized and untreated samples. In particular, the contents of chlorogenic acid, catechin hydrate, caffeic acid, rutin, naringin, resveratrol, and quercetin were markedly increased after treatment. Additionally, five phenolic compounds were evaluated by molecular docking analysis against α-amylase and α-glucosidase, and the strongest binding affinities were observed for naringin (−7.40 kcal/mol) and chlorogenic acid (−7.17 kcal/mol), respectively. These findings demonstrate that machine learning-assisted ultrasound–ohmic processing can effectively improve the antidiabetic and functional properties of hawthorn vinegar.

## 1 Introduction

Hawthorn (*Crataegus tanacetifolia*) *species are important natural plant resources that grow widely in temperate and subtropical climate zones and are notable for their* rich bioactive components and positive effects on health (1). Crataegus fruit is a natural functional food source that stands out due to its rich composition of phenolic compounds, flavonoids, ascorbic acid, and dietary fiber. Thanks to these bioactive components, the fruit has been reported to exhibit antioxidant, antimicrobial, anti-inflammatory, immune-boosting, and anticancer properties (2–4).

Vinegar is a widely consumed fermented product due to its nutritional properties and its significant role in production technology. Its unique aroma and taste profile, functional components, and diverse uses give it an important place in both modern and traditional diets across many cultures (5). Studies show that hawthorn vinegar contains high levels of phenolic compounds and exhibits antioxidant activity, and is rich in bioactive components such as catechin, vanillic acid, gallic acid, epicatechin, caffeic acid, and ellagic acid (6, 7). Among the non-thermal technologies applied to vinegars, ultrasound technology, which has gained popularity today, has become a frequently studied topic in the current literature regarding its positive effects on food components across many products (8, 9).

Response Surface Methodology (RSM) is widely used to present food processing products. RSM is a technique developed by Kleijnen (61) that enables the healthy maintenance of relationships between response and independent variables using decomposed and separated models (10, 11). In recent years, modeling experimental data obtained using RSM with machine learning algorithms has gained increasing interest in the field of food science. Algorithms such as artificial neural networks, support vector machines, gradient boosting, and random forests can model complex, nonlinear relationships with high accuracy (12–14). This hybrid approach combines the systematic structure of experimental design with the powerful predictive capabilities of machine learning, enabling more comprehensive results in process optimization. Indeed, the integration of RSM with machine learning algorithms for ultrasound-assisted bioactive component extraction from grape seeds has achieved high predictive accuracy for optimizing extraction parameters (15). However, in small datasets (*n* < 50), algorithm selection and hyperparameter tuning significantly affect model performance. Therefore, systematic hyperparameter optimization is a prerequisite for developing reliable models in small-scale experimental designs.

Molecular docking is a key computational technique used to predict the preferred way in which a small molecule interacts with a receptor (16). It is widely applied in structural biology and drug discovery. This method also enables the evaluation of potential drug candidates prior to experimental studies, thereby saving time, cost, and labor (17, 18).

While the effects of ultrasound and ohmic heating on various food matrices have been studied separately, their combined applications are quite limited in the literature. To our knowledge, no previous study has successfully integrated advanced regularized machine learning algorithms with meta-heuristic optimization to precisely model and maximize the properties of vinegar treated with US-OH. This study combines Box–Behnken experimental design with machine learning to optimize the inhibitory activities against α-glucosidase and α-amylase during the ohmic-ultrasonication process for hawthorn vinegar. Thirteen different machine learning algorithms were comparatively evaluated on the obtained data using systematic hyperparameter optimization with GridSearchCV. Using the most successful model, the optimum process conditions maximizing the inhibition activity of both enzymes were determined with three different metaheuristic optimization algorithms (Differential Evolution, PSO, and GWO). The study aims to demonstrate the effectiveness of a hybrid modeling approach that combines experimental design and machine learning for processing food bioactives. This study employed the molecular docking technique to examine the molecular interactions with α-glucosidase and α-amylase.

## 2 Materials and methods

### 2.1 Raw material procurement, production and storage of hawthorn vinegar

Hawthorn samples were obtained from a commercial firm operating in Bolu, Türkiye. The fruits were transported to the laboratory in a cold chain, cleaned of foreign matter, and stored under appropriate conditions until the production stage. Unprocessed (control) hawthorn vinegar was prepared using the traditional method of vinegar production, and the production was carried out with a two-stage (acetic acid and ethyl alcohol fermentation). To initiate ethyl alcohol fermentation, *Saccharomyces cerevisiae* was inoculated into the mixture, and the initial inoculation level was adjusted to 10^6^ CFU/mL. To initiate acetic acid fermentation, 5% (v/v) vinegar was added to the mixture. The fermentation process was carried out at 28 °C and continued for up to 65 days. After the completion of this fermentation, the total acidity was 4%, and the ethanol content was 0.5–1.0%. The produced hawthorn vinegar samples were placed in sterile glass bottles and stored at −20 ± 1 °C until analysis and processing.

### 2.2 Ultrasound treatment (US)

Ultrasound treatment (US) was performed in continuous mode using a probe-type ultrasound homogenizer (Hielscher UP200 St, 26 kHz, 200 W; Berlin, Germany). Samples were processed under an ice bath. Ultrasound parameters were defined as amplitude (40, 60, 80%) and duration (2, 4, 6 min).

### 2.3 Thermal pasteurization treatment (TP)

The thermal pasteurization of the samples of hawthorn vinegar was carried out using a temperature-controlled water bath. The hawthorn vinegar samples were transferred into sterile glass bottles and subjected to thermal treatment at 85 ± 1 °C for 2 min. Immediately after pasteurization, the samples were rapidly cooled in an ice-water bath to minimize further thermal effects. The thermally processed samples were coded as TP-HV and stored at −20 ± 1 °C until further analyses.

### 2.4 Ohmic heating application (OH)

**Figure 1:**
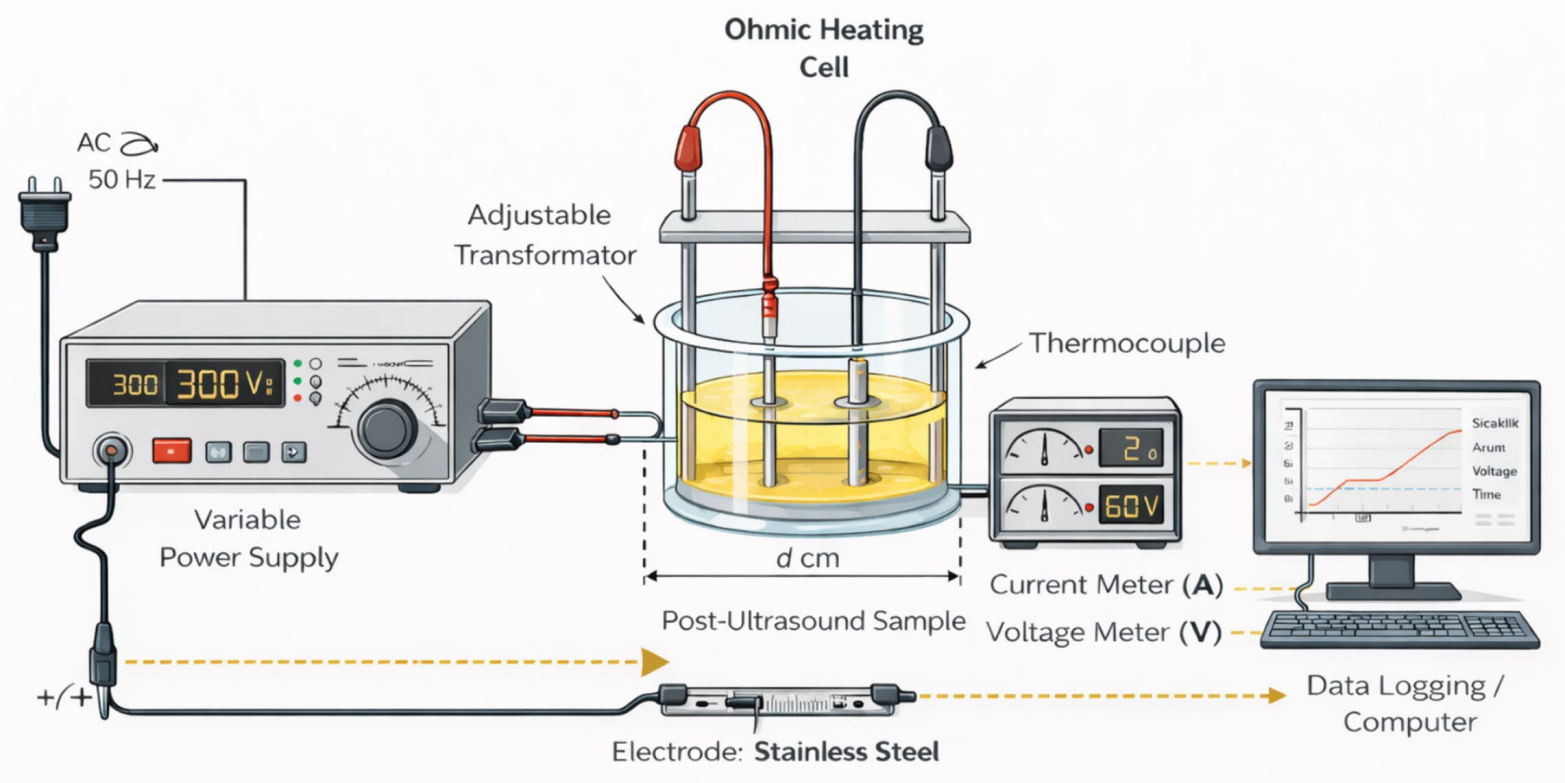
Schematic representation of the ohmic heating process involved in the ultrasound-ohmic combination. The system consists of an AC power supply, electrodes, processing cell, temperature sensor, and data logging unit.

The ohmic heating system consists of an AC power supply, electrodes, and a data logging unit. The distance between electrodes is 6 cm, calculated using E = V/d. Samples were processed in 300 mL volumes, and temperature was monitored using a PT100 sensor. Mixing was performed at 300 rpm. Parameters were applied at 20–40 V/cm and 2–6 min. Ultrasound–ohmic combination procedure (US–OH): The procedure was performed in US→OH order. The system diagram is given in Figure 1.

### 2.5 Data set and preprocessing

The dataset obtained from the experimental design consists of 27 experimental points. Four independent variables (ultrasound amplitude, ultrasound duration, ohmic field strength, and ohmic heating time) were used as inputs in the modeling process; α-glucosidase and α-amylase inhibitor activities were determined as dependent variables and modeled separately. The experimental ranges of the independent variables are presented in Table 1.

**Table 1:** Experimental values for the independent variables in the dataset used.

Variable	Unit	Minimum	Maximum	Mean	Std. Deviation
X_1_: Ultrasound amplitude	%	40	80	60.00	13.59
X_2_: Ultrasound time	min	2	6	4.00	1.36
X_3_: Ohmic field strength	V/cm	20	40	30.00	6.79
X_4_: Ohmic heating time	min	2	6	4.00	1.36

Data standardization was applied to eliminate the negative impact of variables of different scales on model performance. The mean of each feature was set to zero and the standard deviation to one. A cross-validation loop was used to prevent leakage. This approach improves the reliability of the model’s performance predictions by preventing statistical information from the test sample from being incorporated into the training data (19). All 27 individual experimental runs of the Box–Behnken design, including the three replicated center-point runs, were used directly as separate observations during model development; no averaging or aggregation of replicates was performed prior to training.

### 2.6 Machine learning algorithms

In this study, 13 different machine learning algorithms were evaluated comparatively. Considering the decisive effect of algorithm selection and hyperparameter tuning on model performance in small datasets (*n* = 27), systematic hyperparameter optimization was applied to all algorithms using GridSearchCV. The evaluated algorithms are described below.

Three algorithms were examined within the scope of polynomial-based linear models.

- Ridge Regression is an extended version of linear regression with L2 regularization (20). It is effective at preventing overfitting in multicollinearity and on small datasets. In this study, second- and third-degree polynomial features were evaluated separately. The regularization parameter α was optimized over {0.001, 0.01, 0.1, 0.5, 1.0, 5.0, 10.0, 50.0} for the second-degree model and over {0.1, 1.0, 10.0, 50.0, 100.0, 500.0} for the third-degree model.
- Lasso Regression, a method that applies L1 regularization, performs automatic feature selection by reducing the coefficients of insignificant variables to zero (21). The regularization parameter α is optimized over the set {0.0001, 0.0005, 0.001, 0.005, 0.01, 0.05, 0.1, 0.5}.
- ElasticNet Regression is a hybrid approach that uses a combination of L1 and L2 regularization (22). In this method, the regularization parameter α ∈ {0.001, 0.01, 0.1, 0.5} and the L1 ratio ∈ {0.1, 0.3, 0.5, 0.7, 0.9} were optimized together.

Within the scope of kernel-based models, two different algorithms were evaluated.

- Support Vector Regression (SVR) was tested separately with radial basis function (RBF) and polynomial kernels (23). SVM is one of several machine learning algorithms that have been used successfully to optimize non-thermal food processing technologies, including ultrasound, pulsed light, and high-pressure processing (24). For the RBF kernel, the parameters were optimized over C ∈ {0.1, 1, 10, 50, 100}, *ε* ∈ {0.01, 0.05, 0.1, 0.2}, and γ ∈ {scale, auto}; for the polynomial kernel, over C ∈ {0.1, 1, 10, 50}, degree ∈ {2, 3}, and ε ∈ {0.01, 0.1}.
- Gaussian Process Regression (GPR) is a non-parametric method that provides both predictions and measures of uncertainty by offering a probabilistic approach (25). Two different configurations were tested using the RBF and Matérn (*ν* = 2.5) kernels. The noise parameter α was optimized over {10^−4^, 10^−3^, 10^−2^, 0.1, 0.5, 1.0} for the RBF kernel and over {10^−4^, 10^−3^, 10^−2^, 0.1, 0.5} for the Matérn kernel.
  Within the scope of ensemble methods, four algorithms have been examined.
- Random Forest is a community method created by combining multiple decision trees (26). The number of trees ∈ {30, 50, 100}, the maximum depth ∈ {2, 3, 4, 5, None}, and the minimum number of samples per leaf ∈ {1, 2, 3, 5} were optimized.
- Gradient Boosting creates a powerful predictive model by correcting the errors of trees trained sequentially (27). The number of trees ∈ {30, 50, 100}, the maximum depth ∈ {2, 3, 4}, the learning rate ∈ {0.01, 0.05, 0.1, 0.2}, and the minimum number of samples per leaf ∈ {2, 3, 5} were optimized.
- XGBoost is an optimized implementation of the gradient boosting algorithm (28). The number of trees ∈ {30, 50, 100}, the maximum depth ∈ {2, 3, 4}, the learning rate ∈ {0.01, 0.05, 0.1, 0.2}, and the minimum child weight ∈ {1, 3, 5} were optimized.
- LightGBM is a gradient enhancement implementation that utilizes a histogram-based learning strategy (29). The number of trees ∈ {30, 50, 100}, the maximum depth ∈ {2, 3, 4, −1}, the learning rate ∈ {0.01, 0.05, 0.1, 0.2}, and the minimum number of child samples ∈ {3, 5, 10} were optimized.
- Finally, the k-nearest neighbors (KNN) algorithm, an instance-based method that predicts the response as a (distance-weighted) average of the nearest training samples, was also evaluated. The number of neighbors ∈ {3, 5, 7, 9}, the weighting scheme ∈ {uniform, distance}, and the distance metric ∈ {euclidean, manhattan} were optimized.

### 2.7 Model evaluation strategy

In datasets with small sample sizes, the traditional training-test split approach can lead to high variance and unreliable performance estimates due to the limited test set. Therefore, a 5-fold cross-validation strategy was preferred in model evaluation in this study.

A nested validation structure has been adopted for hyperparameter optimization. GridSearchCV is used to perform a systematic search over the hyperparameter space defined for the model. The combination of parameters that yields the maximum cross-validation R^2^ is then selected. This approach prevents optimistic performance estimations by ensuring that hyperparameter selection is also performed within the cross-validation framework. All hyperparameters were tuned via an exhaustive grid search (GridSearchCV) with 5-fold cross-validation, using the coefficient of determination (R^2^) as the scoring criterion. The K-Fold splitter was applied with shuffling enabled and a fixed random seed (random_state = 42), and the same seed was used for all stochastic estimators to ensure full reproducibility of the machine learning workflow.

During the cross-validation stage, the dataset was split into five equally sized parts. For each iteration, four sections were selected for model training and one section for model validation. This whole sequence was performed five times. The final performance metrics were expressed as the averages and standard deviations of the five folds. The main advantages of cross-validation are:

- using all data for both training and validation,
- reducing the variance of performance estimates, and
- evaluating the consistency of the model across different data subsets (30).

In this study, the level of overfitting was also monitored by calculating the difference between the training and validation R^2^ values (Train-CV Gap).

### 2.8 Performance metrics

In evaluating model performance, the root mean square error (RMSE), mean absolute percentage error (MAPE), coefficient of determination (R^2^), and mean absolute error (MAE) were used. The R^2^ metric represents the explanatory power of the model, RMSE and MAE represent the absolute prediction error, and MAPE represents the percentage magnitude of the errors. In addition to these metrics, the difference between the training and cross-validation R^2^ values (Train-CV Gap) was calculated to monitor the models’ generalization capacity and level of overfitting. Low gap values indicate that the model is not overfitting the training data.

### 2.9 Optimization

In this study, optimum process conditions that maximize α-glucosidase and α-amylase inhibitor activities were determined using the best-performing model. During the optimization process, the model exhibiting the highest cross-validation performance was used as the objective function. The optimization algorithms queried the model for each candidate solution (process parameter combination) in the search space, obtained the predicted response value, and iteratively searched for the best solution by maximizing this value. This approach enabled the rapid and reliable evaluation of thousands of parameter combinations that cannot be experimentally tested. Three different metaheuristic algorithms were used comparatively in the optimization process.

- Differential Evolution (DE) is an evolutionary algorithm developed for global optimization in continuous space (31). It is preferred because it does not require gradient information, can find the global optimum in multimodal functions without getting stuck on local minima, and can work with a small number of control parameters.
- Particle Swarm Optimization (PSO) draws inspiration from the collective behavior of bird flocks and fish groups to create a population-based optimization algorithm (32). Each particle scans the search space by moving toward its own optimal position and the swarm’s global optimal position. The configuration is based on an inertial weight of w = 0.72, cognitive and social acceleration coefficients of c₁ = c₂ = 1.49, and a particle count of 40.
- Grey Wolf Optimization (GWO) is a metaheuristic algorithm that draws inspiration from the hunting behavior of grey wolves (33). The wolf pack mimics the mechanisms of encircling and attacking prey, led by alpha, beta, and delta individuals. The wolf population was estimated at 40.

Optimization was performed for three different scenarios:

- Maximizing α-glucosidase inhibition: Maximizing only the α-glucosidase inhibitory activity
- Maximizing α-amylase inhibition: Maximizing only the α-amylase inhibitory activity
- Multiple response optimization: The Desirability function was used to simultaneously maximize both responses. In this approach, the geometric mean of the normalized responses was defined as the objective function.

Optimization was performed within the limits of the experimental design (ultrasound amplitude: 40–80%, ultrasound duration: 2–6 min, ohmic field strength: 20–40 V/cm, ohmic heating time: 2–6 min).

### 2.10 Molecular docking method

The docking performances against α-amylase (PDB ID: 4w93) (34), α-glucosidase (PDB ID: 5nn8) (35) which were downloaded from RCSB protein data bank^1^ with AutoDockTools 4.2 (36). Kollman charges were assigned to the target molecules, considering only polar hydrogen atoms, and all water molecules were removed from the macromolecular structures. The genetic algorithm was implemented using the Lamarckian Genetic Algorithm with a population size of 150. Docking simulations were initiated with randomized ligand positions, and Gasteiger charges were applied to the ligands (37). All visuals were produced in Discovery Studio 4.1.0.

### 2.11 Antidiabetic activity

Using acarbose as a positive control, the antidiabetic potential of hawthorn vinegar samples was assessed by evaluating their inhibitory effects on α-glucosidase and α-amylase. Enzyme inhibition analyses were performed using a modified enzymatic method, while absorbance measurements were taken using an SP-UV/VIS-300SRB UV–Vis spectrophotometer (Spectrum Instruments, Australia) (38).

### 2.12 Individual phenolic compounds (HPLC–DAD)

**Figure 2:**
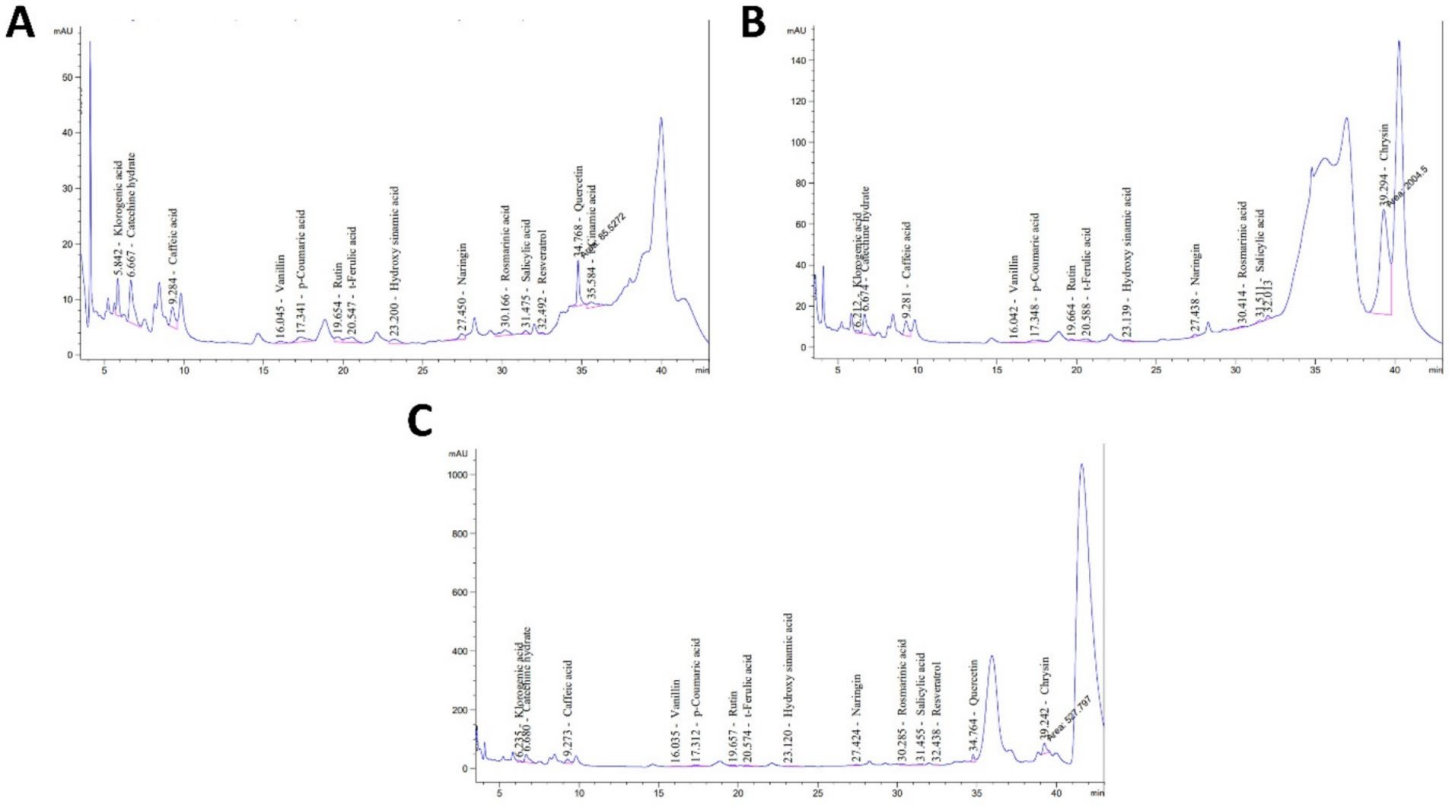
Representative HPLC–DAD chromatograms of individual phenolic compounds in hawthorn vinegar samples: **(A)** CON-HV (control, untreated), **(B)** TP-HV (thermally pasteurized), and **(C)** USOH-HV (ultrasound–ohmic treated). Peak annotations indicate the phenolic compounds that have been identified, as determined by their retention times and UV–Vis spectra, and by comparison with authentic standards.

Individual phenolic compounds in hawthorn vinegar were analyzed in the control (CON-HV; untreated), thermally pasteurized (TP-HV), and ultrasound–ohmic treated (USOH-HV) samples. Representative HPLC–DAD chromatograms of the three sample groups are presented in Figure 2. Prior to HPLC injection, vinegar samples were clarified to remove suspended solids by centrifugation (10 min at 6,000–10,000 × g) and subsequently filtered through a 0.45 μm membrane syringe filter. The separation was performed on an ACE Genix C18 column (250 × 4.6 mm, 5 μm) using an Agilent 1260 HPLC system. The column oven was at 30 °C, the flow rate of the mobile phase was 0.80 mL/min, and the injection volume was 10 μL. The mobile phase was a mixture of water containing 0.1% phosphoric acid and organic solvent (39). DAD detection was conducted at 360, 320, and 280 nm. Phenolic compounds were identified using authentic standards. In determining the LOD and LOQ values for the analytical parameters, signal-to-noise (S/N) ratios of 3 and 10 were used, respectively. To test the accuracy of the method, a standard addition (spike-recovery) procedure was applied at three concentration levels (low, medium, and high; *n* = 3), and the resulting data were expressed as percentage recovery. Intra-system consistency (intra-day accuracy) was measured using successive injections (*n* = 3) and reported as the percentage relative standard deviation (%RSD) of peak areas and concentrations. Quantification was conducted based on external calibration curves, with the resulting data given in μg/mL. Compounds detected as “not detected” were below the method’s threshold. All determinations were triplicate, and results were reported as mean ± SD.

### 2.13 Software and libraries

All analyses were based on the Python 3.x programming language. The development of machine learning models and the optimization of hyperparameters was achieved by utilizing the scikit-learn library (19). The xgboost (28) was chosen for the XGBoost algorithm, the lightgbm package (29) for the LightGBM algorithm, and the optimize module of the SciPy library for optimization operations. The PSO and GWO algorithms were developed specifically for this purpose. Data processing was performed using the pandas and NumPy libraries, while visualization was carried out using the matplotlib and seaborn libraries.

### 2.14 Statistical analysis

One-way ANOVA was used to evaluate the differences between the treatment groups, followed by a Tukey’s HSD *post hoc* test. All experiments were expressed as mean ± SD and performed in triplicate. Differences were considered statistically significant at *p* < 0.05.

For machine learning, the activities of α-glucosidase and α-amylase were defined as response variables, with ultrasound amplitude, treatment time, ohmic electric field strength, and ohmic heating time used as input variables. The dataset was standardized during cross-validation to prevent data leakage. Model performance was evaluated using 5-fold cross-validation, with predictive accuracy assessed based on RMSE, MAPE, MAE, R^2^, and Train-CV Gap.

Hyperparameter optimization was done using GridSearchCV. The best model was selected based on cross-validation R^2^ and prediction error. This was then used to determine the optimum conditions. This was done by single-response, then multiple-response, optimization to maximize α-glucosidase and α-amylase inhibitory activities. The desirability function was used to optimize both simultaneously.

## 3 Results and discussion

### 3.1 Model comparison

In this study, the performance of 13 machine learning algorithms for predicting α-amylase and α-glucosidase inhibitory activity was evaluated using GridSearchCV with hyperparameter optimization and 5-fold cross-validation. The results from the models for α-glucosidase inhibition prediction are presented in Table 2, and those for α-amylase inhibition prediction in Table 3.

**Table 2:** Results of 5-fold cross-validation for α-glucosidase inhibition prediction.

Model	CV R^2^	CV RMSE	CV MAE	MAPE (%)	Train-CV Gap
Lasso Poly2	0.9301	0.3845	0.3280	0.95	0.0580
ElasticNet Poly2	0.9288	0.3906	0.3267	0.94	0.0581
Ridge Poly2	0.9267	0.3958	0.3533	1.02	0.0590
SVR (RBF)	0.7680	0.7155	0.5601	1.62	0.2320
LightGBM	0.7046	0.7970	0.6749	1.95	0.2440
Gradient boosting	0.6461	0.8583	0.7358	2.16	0.3060
Ridge Poly3	0.6489	0.8364	0.7041	1.96	0.3470
XGBoost	0.5945	0.9495	0.7484	2.21	0.4010
Gaussian process	0.5701	0.9623	0.7695	2.24	0.4300
GPR (Matern)	0.4923	1.0467	0.8512	2.47	0.5080
Random forest	0.4964	1.0501	0.8944	2.58	0.4340
KNN	0.4538	1.0418	0.7748	2.27	0.5460
SVR (Poly)	0.2963	1.2103	0.9862	2.77	0.4480

CV RMSE, cross-validation root mean square error; CV R^2^, cross-validation coefficient of determination; CV MAE, cross-validation mean absolute error; SVR, support vector regression; MAPE, mean absolute percentage error; Train-CV Gap, difference between training and cross-validation performance; RBF, radial basis function; LightGBM, Light Gradient Boosting Machine; XGBoost, Extreme Gradient Boosting; GPR, Gaussian process regression; KNN, k-nearest neighbors; Poly2, second-degree polynomial; Poly3, third-degree polynomial.

**Table 3:** Results of 5-fold cross-validation for α-amylase inhibition prediction.

Model	CV R^2^	CV RMSE	CV MAE	MAPE (%)	Train-CV Gap
Lasso Poly2	0.9299	0.3861	0.3341	0.92	0.0630
ElasticNet Poly2	0.9292	0.3882	0.3328	0.91	0.0640
Ridge Poly2	0.9225	0.4118	0.3597	0.98	0.0700
SVR (RBF)	0.7726	0.7586	0.5954	1.61	0.2270
LightGBM	0.7150	0.8200	0.7041	1.93	0.2210
Gradient boosting	0.6038	0.9576	0.7975	2.17	0.3570
XGBoost	0.6033	1.0084	0.7874	2.15	0.3690
Gaussian process	0.5950	1.0174	0.8288	2.26	0.405
Ridge Poly3	0.5876	0.9749	0.8958	2.35	0.4090
GPR (Matern)	0.5346	1.0918	0.9055	2.45	0.4650
KNN	0.4482	1.1308	0.8404	2.31	0.5520
Random forest	0.4395	1.1913	1.0222	2.76	0.4820
SVR (Poly)	0.3226	1.2375	1.0313	2.72	0.4820

CV RMSE, cross-validation root mean square error; CV R^2^, cross-validation coefficient of determination; CV MAE, cross-validation mean absolute error; Train-CV Gap, difference between training and cross-validation performances; MAPE, mean absolute percentage error; SVR, support vector regression; LightGBM, Light Gradient Boosting Machine; RBF, radial basis function; XGBoost, Extreme Gradient Boosting; GPR, Gaussian process regression; KNN, k-nearest neighbors; Poly2, second-degree polynomial; Poly3, third-degree polynomial.

**Figure 3:**
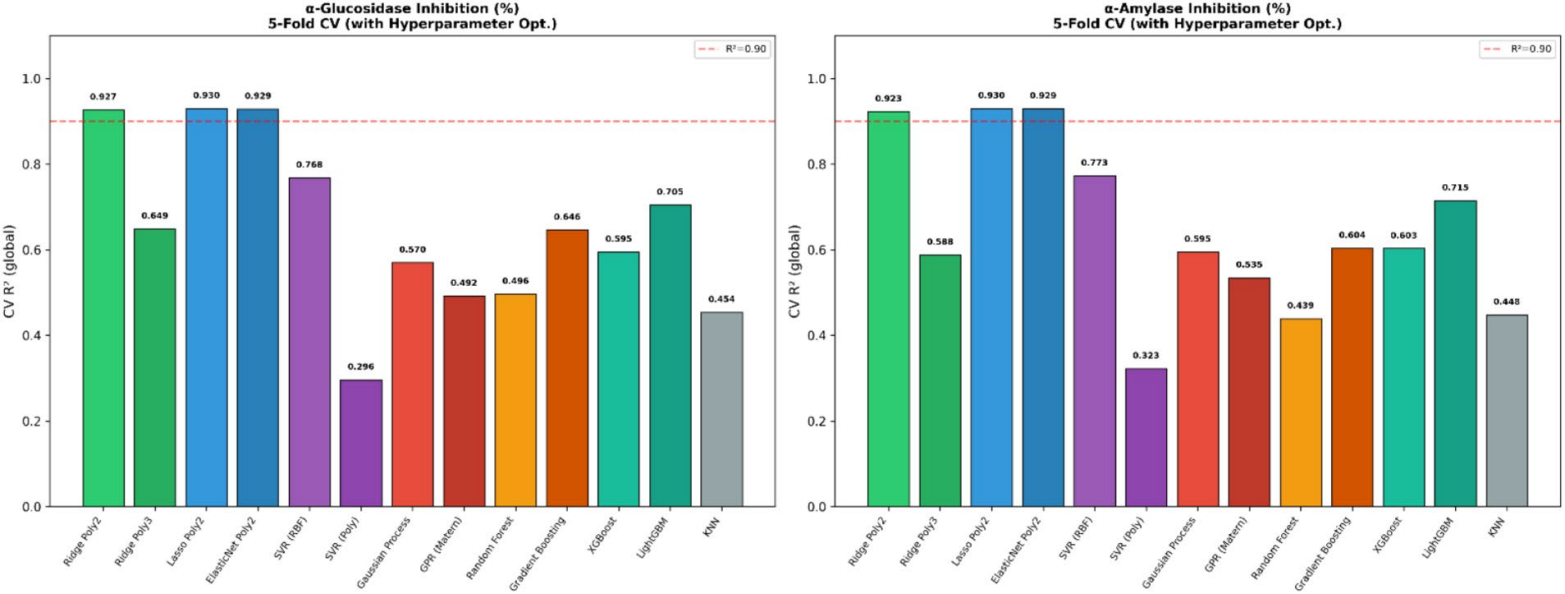
Model-based comparison of *R*^2^ values obtained with 5-fold cross-validation.

Examining the results shown in Tables 2, 3, it is evident that polynomial-based linear models (Lasso, ElasticNet, Ridge) exhibit significantly superior performance compared to all other algorithm classes. The Lasso Poly2 model achieved the highest CV R^2^ values for both dependent variables (α-glucosidase: 0.9301, α-amylase: 0.9299). This model was followed by ElasticNet Poly2 (0.9288/0.9292) and Ridge Poly2 (0.9267/0.9225), respectively. The model comparison is visualized in Figure 3. In Figure 3, the left panel shows the α-glucosidase predictions, and the right panel shows the α-amylase predictions. Error bars represent the standard deviation.

The MAPE values of the Lasso Poly2 model were calculated as 0.95% for α-glucosidase and 0.92% for α-amylase. These values indicate that the model’s mean prediction error is below 1% and that it achieves high accuracy in practical applications. The success of Lasso Regression is based on several key factors. The first is the inclusion of second-degree polynomial features. This allows for the capture of nonlinear relationships between independent and dependent variables. The second factor is that the L1 regularization mechanism both prevents overfitting and performs automatic feature selection. Lasso’s L1 penalty term reduces model complexity by reducing the coefficients of insignificant polynomial terms to zero (21). The third and decisive factor is the optimization of the regularization parameter (α) with GridSearchCV. The best-performing Lasso Poly2 model consisted of a scikit-learn Pipeline combining standardization, second-degree polynomial feature expansion (PolynomialFeatures, degree = 2, without bias term), and a Lasso estimator (max_iter = 10,000). The regularization parameter α, optimized via GridSearchCV, was determined as α = 0.005 for α-glucosidase and α = 0.01 for α-amylase. These low regularization values resulted in a significant performance improvement over models with fixed parameters. The fact that the Train-CV Gap value remained only around 0.06 confirms the effectiveness of these mechanisms.

When other models were examined, it was determined that the SVR (RBF) and LightGBM algorithms showed moderate performance (*R*^2^ ≈ 0.70–0.77), while the Random Forest, XGBoost, Gaussian Process, and KNN algorithms exhibited low performance (*R*^2^ < 0.60). The low performance of the other machine learning algorithms mentioned can be explained by different reasons. The SVR and Gaussian Process algorithms did not learn sufficient patterns from the small dataset and exhibited high variance (Train-CV Gap > 0.22). Ensemble methods such as Random Forest, XGBoost, LightGBM, and Gradient Boosting generally show superior performance in large datasets. These algorithms require a sufficient number of samples to work effectively; a dataset of 27 samples was insufficient for these algorithms to learn complex patterns. From a bias–variance perspective, this outcome is expected: with only 27 observations, flexible high-capacity learners (ensemble and kernel-based methods) tend to fit noise and exhibit high variance, whereas regularized second-degree polynomial models impose a strong yet appropriate structural prior on the response surface. Because Box–Behnken designs are inherently constructed to be modeled by second-order polynomial functions, the polynomial-based learners are well matched to the underlying data-generating structure, and their L1/L2 regularization further suppresses overfitting. This combination of an appropriate functional form and effective regularization explains their superior and more stable performance in this small-sample regime.

### 3.2 Model validation

**Figure 4:**
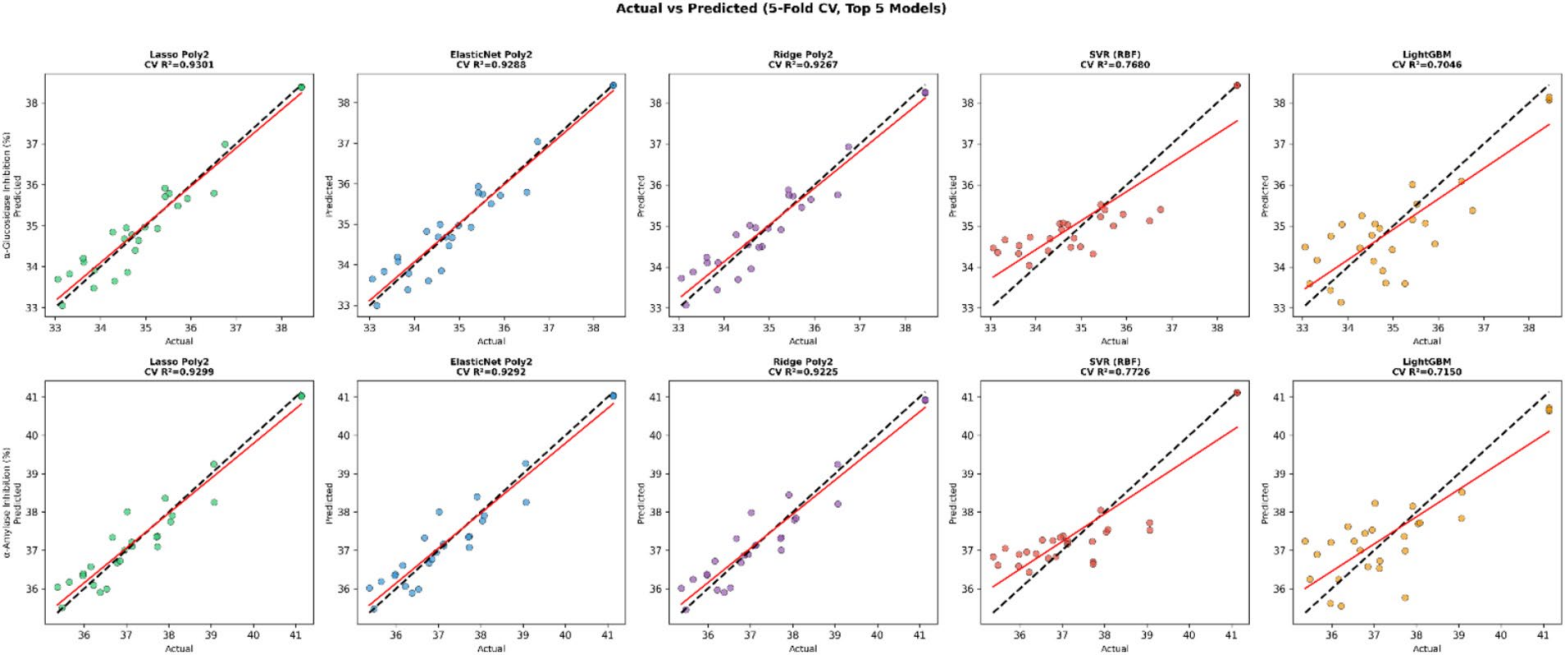
Comparison of actual and predicted values obtained through cross-validation (top 5 models).

After the tests were conducted, the prediction accuracy of the Lasso Poly2 model, which performed best, was evaluated by comparing actual and predicted values. Figure 4 presents the actual-prediction graphs of the top five models. It is observed that the Lasso Poly2 model predicts the data points very close to the 1:1 line. In other models, significant deviations are observed, especially at high and low values.

**Figure 5:**
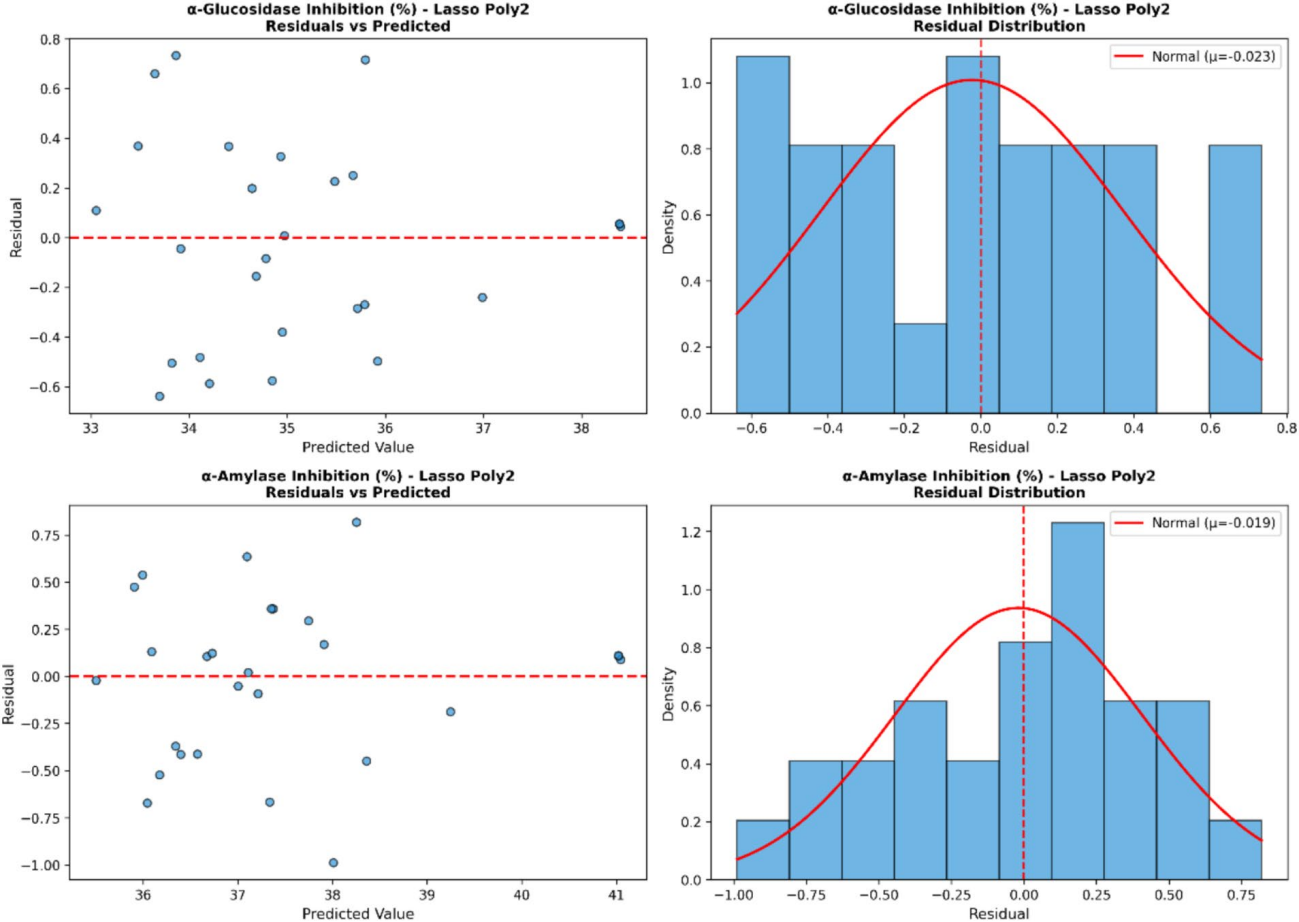
Residual analysis for the Lasso Poly2 model. The left column shows residual-prediction plots, and the right column shows residual histograms.

The residual analysis for the Lasso Poly2 model is presented in Figure 5. The plot of residuals against predicted values shows no systematic pattern; the residuals are randomly distributed around zero. This indicates that the model successfully captures nonlinear relationships and does not contain a systematic error. Examination of the residual histograms reveals that the distribution is close to normal and is symmetric about zero.

**Figure 6:**
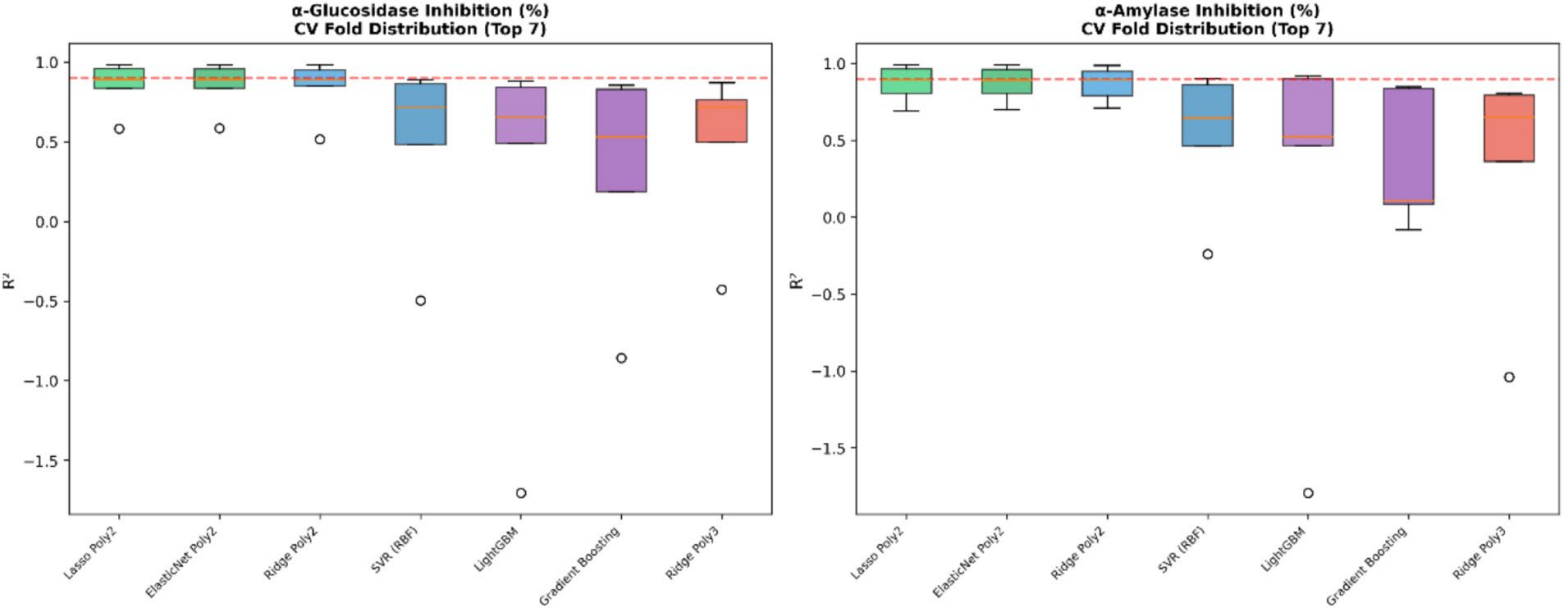
Box plot representation of the fold-based R^2^ scores of the seven best models.

The distribution of fold-based R^2^ scores from the 5-fold cross-validation is shown in the boxplot in Figure 6. The Lasso Poly2 model exhibited consistent performance across all folds, remaining within a narrow score range. In other models, high inter-fold variance was observed, indicating that these models cannot provide reliable performance on small datasets.

### 3.3 Model stability and reliability

**Figure 7:**
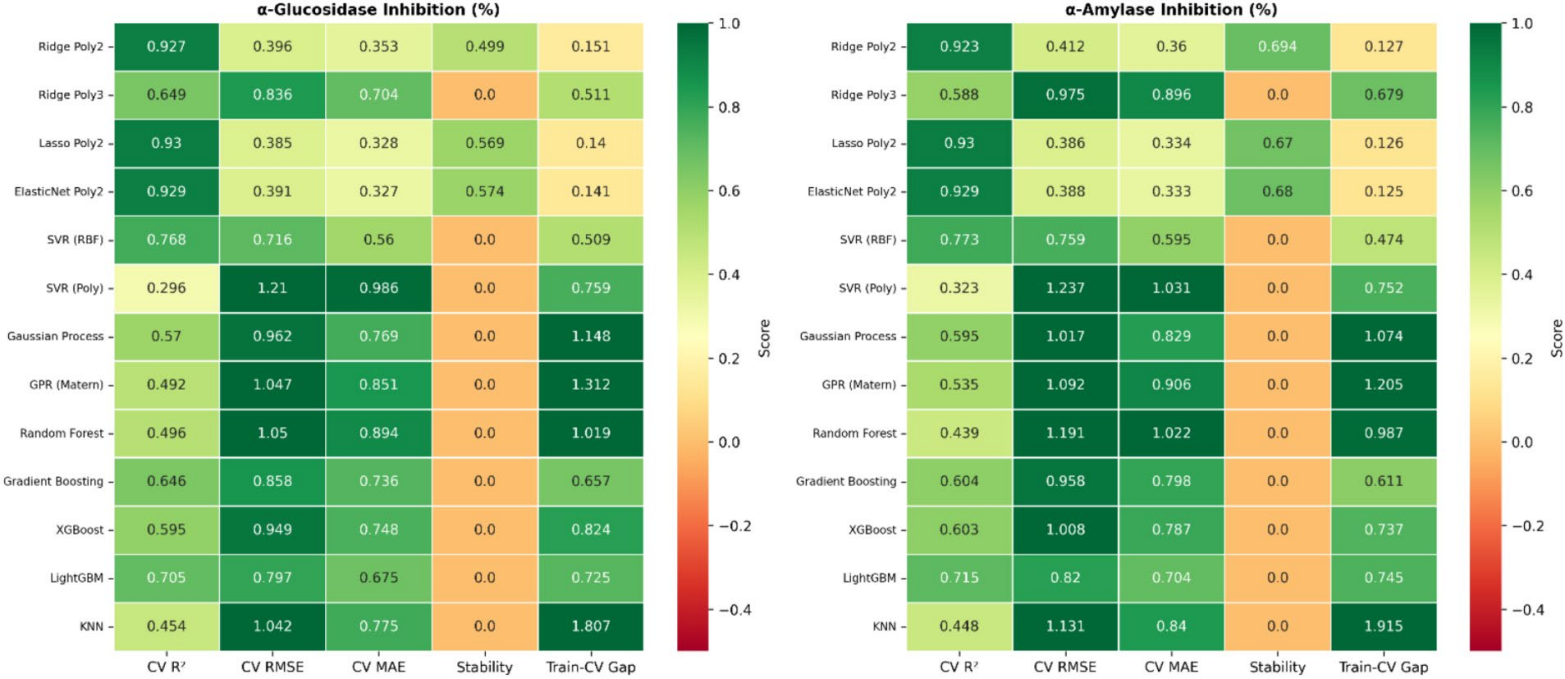
Model performance heat map.

The stability and reliability of the models used in this study were evaluated across multiple metrics. The performance heatmap in Figure 7 presents a comparative analysis of the CV R^2^, CV RMSE, CV MAE, Stability, and Train-CV Gap metrics for all models. The Lasso Poly2 model consistently performs best across all metrics.

The reliability of machine learning models is determined by their ability to generalize to new data, rather than their performance on training data. It was found that in this study, the risk of overfitting was minimized using two main strategies. The first strategy is the use of the scikit-learn Pipeline structure. Data standardization and model training are encapsulated in a single pipeline, and during cross-validation, the standardization parameters (mean and standard deviation) for each fold are calculated only from that fold’s training data. This approach prevents data leakage, ensuring realistic performance predictions. Data leakage occurs when information contained in test data interferes with the training stage, resulting in overly optimistic performance predictions (19).

The second strategy is to use 5-fold cross-validation. Traditional training-test splits (70–30% or 80–20%) can produce unreliable results on small datasets because a small number of samples in the test set leads to high variance. Cross-validation solves this problem by ensuring that all samples are used for both training and validation purposes (30).

The Train-CV Gap metric was used to assess overfitting. This metric represents the difference between the training and cross-validation R^2^ values. The Train-CV Gap value for the Lasso Poly2 model was calculated as 0.063 for α-amylase and 0.058 for α-glucosidase. These low values indicate that the model is not overfitting the training data and can generalize to new data. In contrast, the Train-CV Gap values for other models ranged from 0.22 to 0.55, indicating significant overfitting. A four-factor Box–Behnken design was used to generate the dataset for this study, comprising 27 experimental runs. This number corresponds to the standard run count of the Box–Behnken configuration for four independent variables and represents a deliberate balance between experimental feasibility and adequate coverage of the design space. Nevertheless, the limited sample size imposes constraints on the model’s generalizability. Although the adopted strategy—systematic hyperparameter optimization, leakage-free Pipeline construction, and 5-fold cross-validation—was specifically designed to obtain reliable performance estimates under these conditions, the resulting models are primarily valid within the investigated experimental domain and should be extrapolated beyond these boundaries only with caution. The superior performance of regularized polynomial models over more complex algorithms is itself partly a consequence of the small sample size, as flexible learners such as ensemble and kernel-based methods typically require larger datasets to realize their full potential. Future studies could expand the experimental dataset, either through additional design points or sequential data augmentation, to further improve model robustness, enable the use of higher-capacity algorithms, and broaden the applicability of the predictive framework.

**Figure 8:**
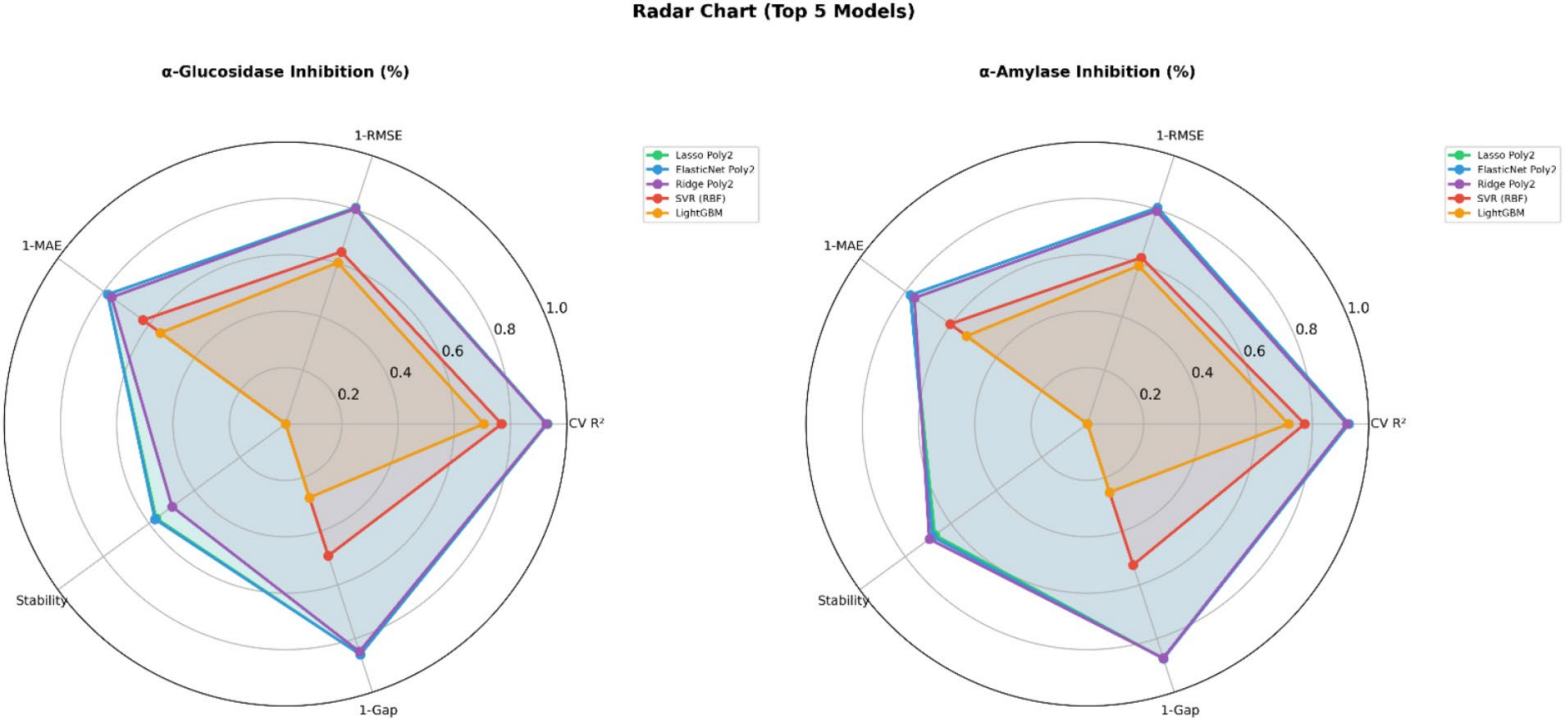
Radar graph for comparing models across multiple performance dimensions.

The radar graph in Figure 8 compares the models across five performance dimensions (CV R^2^, 1-RMSE, 1-MAE, Stability, 1-Gap). The Lasso Poly2 model is clearly distinguished from the other models by achieving values close to the outer circle in all dimensions.

**Figure 9:**
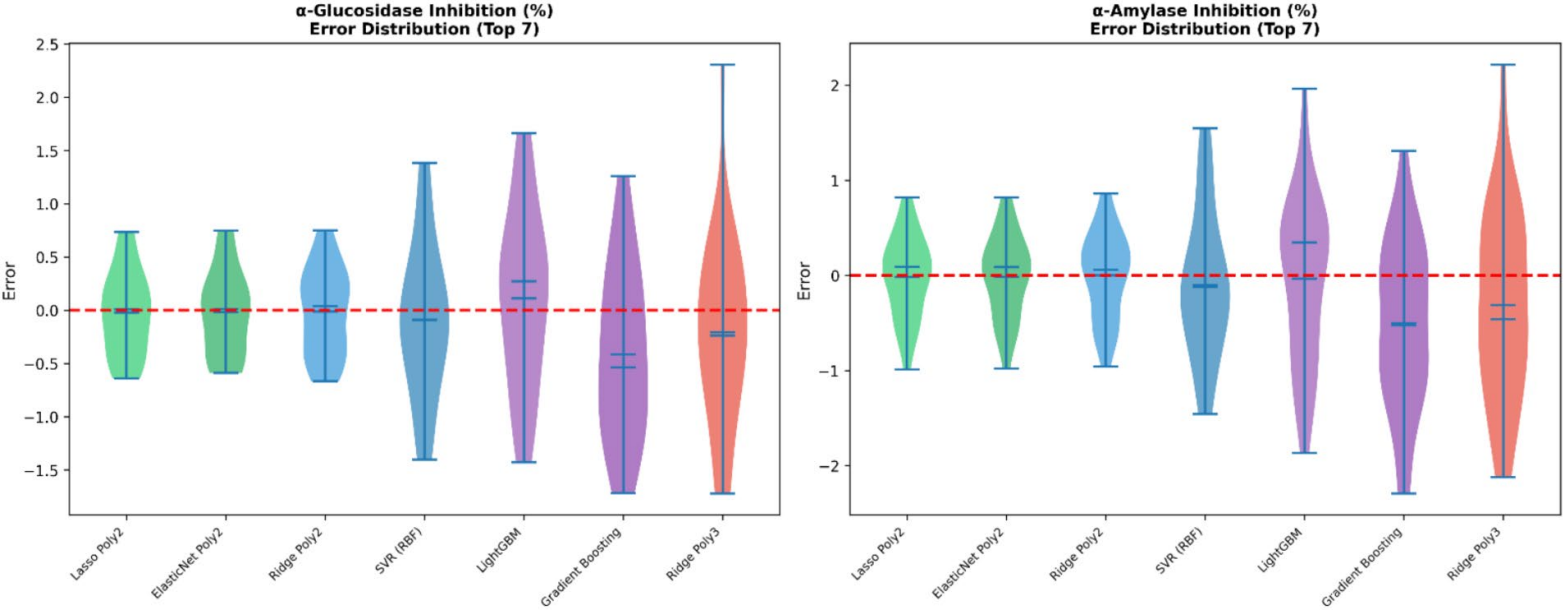
Representation of prediction errors using a violin graph.

The distribution of prediction errors is shown in the violin plot in Figure 9. The Lasso Poly2 model has the narrowest error distribution, with the majority of errors concentrated at values close to zero. In other models, the error distributions are wider and exhibit an asymmetrical structure.

### 3.4 Variable importance analysis

**Figure 10:**
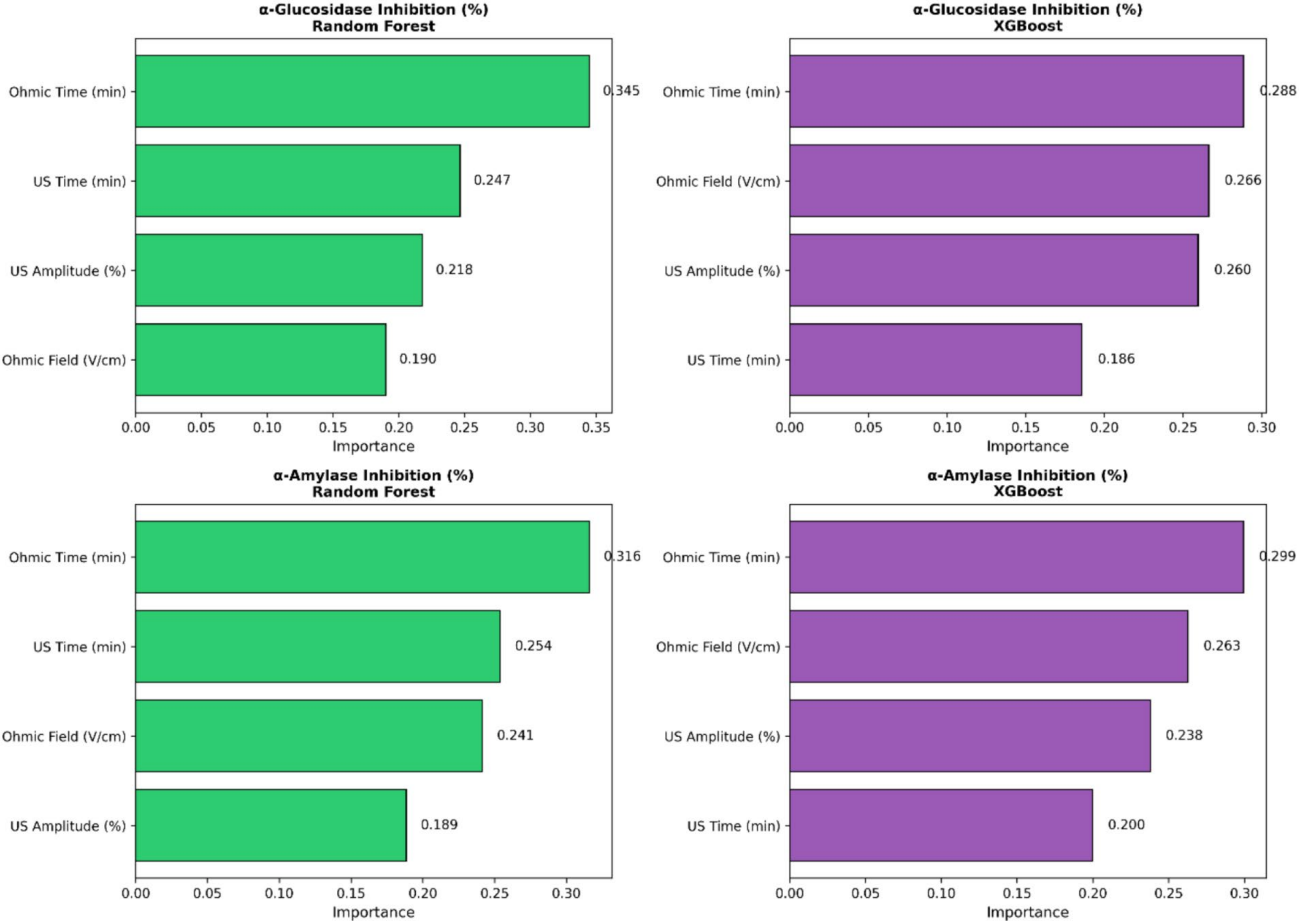
Variable significance analysis based on Random Forest and XGBoost algorithms.

The relative influence of independent variables on the dependent variables was evaluated using the impurity-based feature importance scores of the Random Forest and XGBoost algorithms. Although these tree-based models did not achieve the predictive accuracy of the Lasso Poly2 model, they were deliberately selected for the importance analysis because they provide intrinsic, model-agnostic importance measures defined directly on the original input variables. In contrast, the best-performing Lasso Poly2 model operates on an expanded set of second-degree polynomial terms (including interaction and quadratic features), so its coefficients are distributed across transformed features and do not translate directly into a single interpretable importance value per original variable. Tree-based importance scores therefore offer a more transparent and directly interpretable assessment of the relative contribution of each process parameter, and are used here as a complementary interpretive tool rather than as predictive models. Examining the results presented in Figure 10, similar patterns are observed for both algorithms and both dependent variables.

The most effective variable for predicting α-glucosidase and α-amylase inhibition was the ohmic heating time (X₄). This was followed by ultrasound amplitude (X₁). Ultrasound duration (X₂) and ohmic field strength (X₃) had relatively lower significance scores.

### 3.5 Optimization results

#### 3.5.1 Single response optimization

Using the Lasso Poly2 model, which demonstrated the best performance in the machine learning process, the optimal process conditions that maximize α-amylase and α-glucosidase inhibitor activities were determined using three different metaheuristic algorithms (DE, PSO, GWO). In other words, the Lasso Poly2 model was used as a cost function within these optimization algorithms. The results of the optimization and multiple-response optimization are shown in Table 4.

**Table 4:** Optimum process conditions maximizing α-glucosidase and α-amylase inhibitor activities.

Parameter	α-Glucosidase Max.	α-Amylase Max.	Combined optimum
X₁: Ultrasound amplitude (%)	64.07	63.21	62.96
X₂: Ultrasound time (min)	4.54	4.45	4.54
X₃: Ohmic field strength (V/cm)	30.89	31.13	30.18
X₄: Ohmic heating time (min)	4.33	4.29	4.27
Predicted α-glucosidase inhibition (%)	38.5534	38.5464	38.5408
Predicted α-amylase inhibition (%)	41.1659	41.1734	41.1491
Experimental Max. α-glucosidase (%)	–	–	37.54 ± 0.53
Experimental Max. α-amylase (%)	–	–	39.27 ± 1.36
Deviation α-glucosidase (%)	–	–	2.60
Deviation α-amylase (%)	–	–	4.57

**Figure 11:**
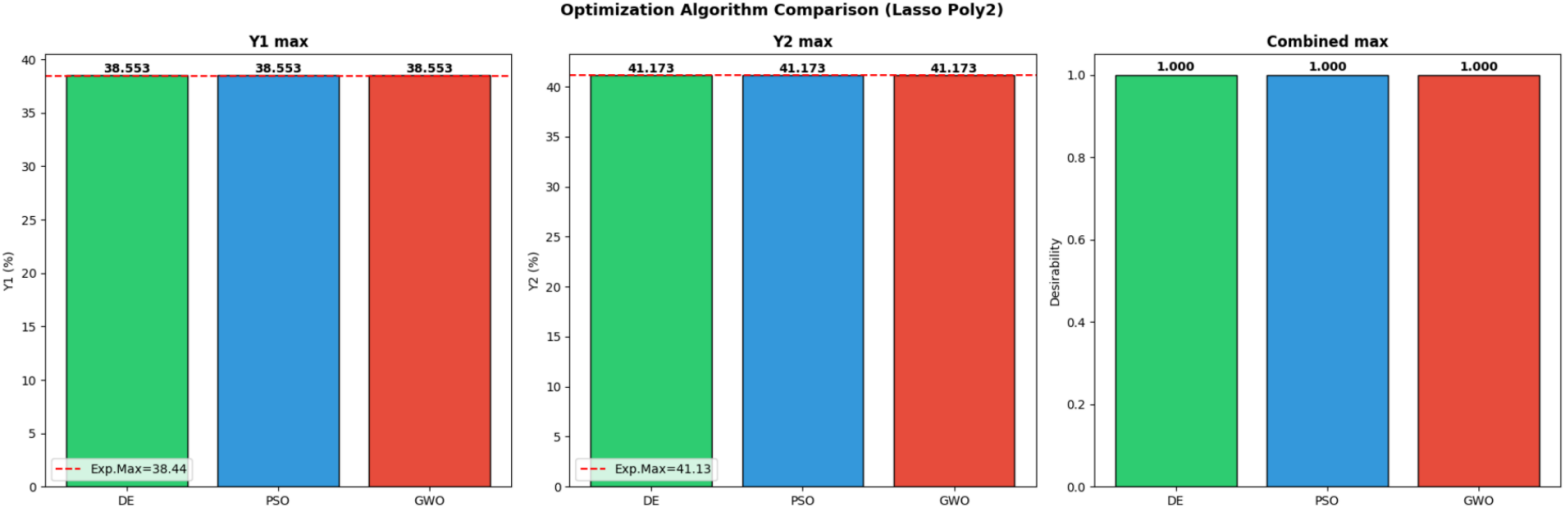
Comparative results of optimization algorithms.

Examining the optimization results shows that the optimal conditions for maximizing α-glucosidase and α-amylase yield very similar values. This shows that the inhibition activity of both enzymes can be maximized under similar process conditions and that, in practice, a single optimum point can satisfy both objectives. The high correlation between the two dependent variables (*r* = 0.9826) supports this finding. The three optimization algorithms (DE, PSO, GWO) converged to practically the same optimum point. This confirms that the response surface has a single well-defined peak and that the obtained optimum is free from the risk of getting stuck in a local optimum. Figure 11 shows the comparative results of the optimization algorithms.

Multiple response optimization was performed using the Desirability function to simultaneously maximize both dependent variables. The combined optimum values presented in the last column of Table 4 are almost identical to the single optimization results. The near-identical optimum conditions for both dependent variables represent a significant practical advantage. This finding demonstrates that α-glucosidase and α-amylase inhibitor activities are affected by similar mechanisms and that a single optimum process condition can satisfy both objectives. Validation was performed under the combined optimal conditions determined by optimization (X₁ = %63.0, X₂ = 4.54 min, X₃ = 30.2 V/cm, X₄ = 4.27 min). Experimental validation results are also presented in Table 4.

Deviations of 4.57% for α-amylase inhibition and 2.60% for α-glucosidase inhibition were observed between model predictions and experimental validation results. These deviations, when considered in the context of experimental uncertainty, support the validity of the model. Considering the standard deviation values observed in the experimental replicates (α-glucosidase: ±0.53, α-amylase: ±1.36), the experimental confidence interval is closely matched by the model predictions. The model prediction for α-glucosidase inhibition (38.54) is 1.22% from the upper confidence limit of the experimental result (37.54 + 0.53 = 38.07); and the model prediction for α-amylase inhibition (41.15) is 1.27% from the upper confidence limit (39.27 + 1.36 = 40.63). These findings are acceptable for a model trained on a small dataset of 27 samples and demonstrate that it points to the correct optimal region.

**Figure 12:**
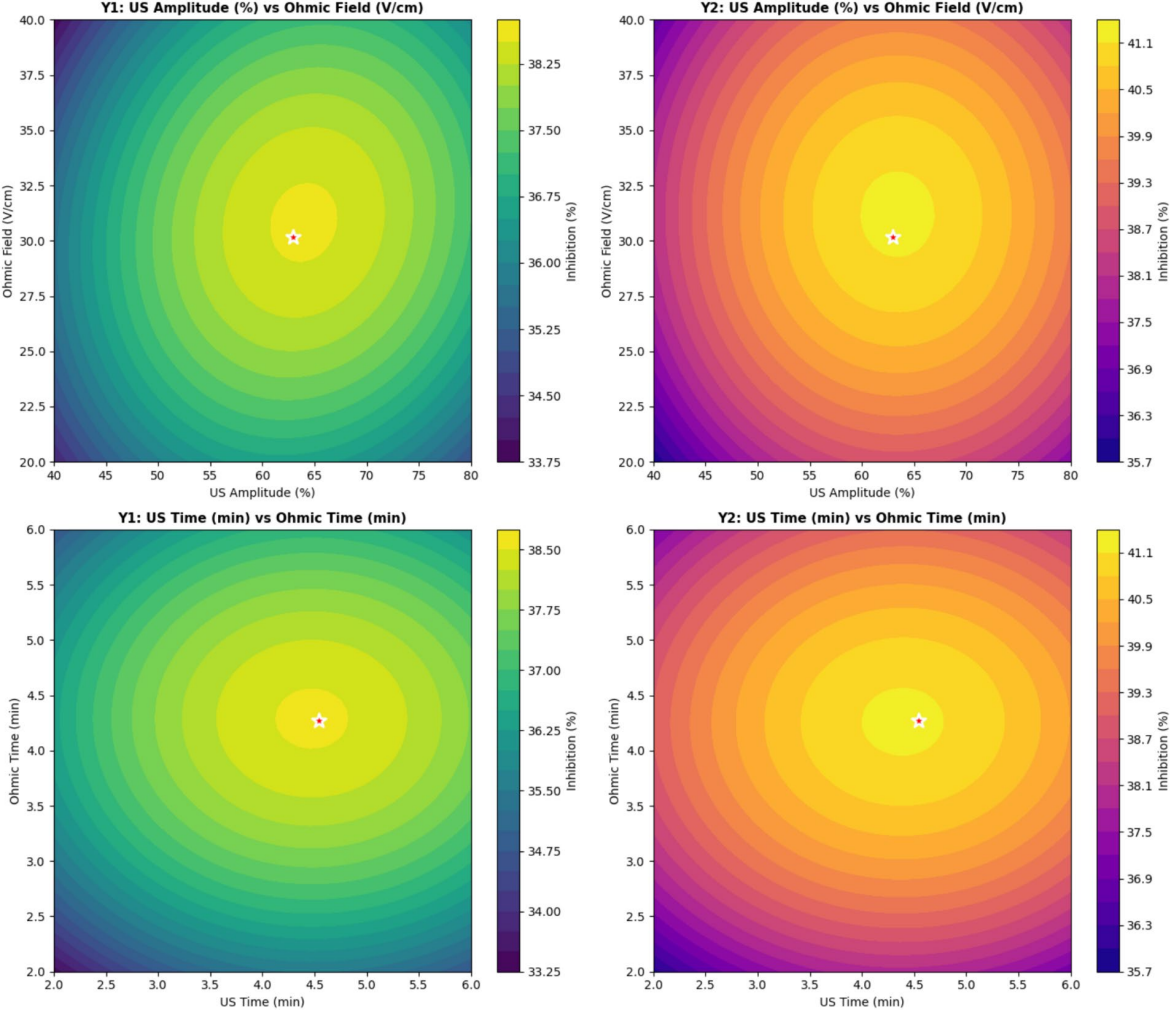
Response surface contour graphs.

The contour plots in Figure 12 provide a two-dimensional visualization of the response surfaces. The upper panel shows the interaction between ultrasound amplitude and ohmic field strength, while the lower panel shows the interaction between ultrasound duration and ohmic heating time. The contour plots confirm that the optimum point (indicated by a red star) is located in the maximum region of the response surface.

### 3.6 Molecular docking

Molecular docking is a computational approach that enables the virtual screening of potential ligands and provides detailed insights into the types and strengths of interactions between a ligand and its target receptor. It is widely used in drug design studies because it facilitates assessment of the structural effects of ligands. Molecular docking results give supportive insights into the possible interaction mechanism of bioactive molecules. Consequently, the appropriateness of the selected target molecule has a key role in determining the reliability of the obtained results (40).

Rawal et al. studied α-amylase inhibitory activity of *Smallanthus sonchifolius* Leaves extract and determined the α-amylase interactions of phytoconstituents by molecular docking method. They recorded the binding affinity ranging from −6.6 to −7.5 kcal/mol while Caffeic acid has −6.6 kcal/mol binding values and Chlorogenic acid has −7.5 kcal/mol with the residue including Asp197, Asp300, His299, and Glu233 (41). In another recent study, Sireesha and Prasad analyzed antidiabetic potentials of N-2-aryl-1,2,3-triazoles type molecules with *in-vitro* methods and performed molecular docking methods against α-amylase for two of their molecules. They recorded H-bonds and pi interactions with residues including Asp197, His101, Leu162, Glu233, Arg195, and His299 (42). Arora et al. investigated the α-amylase inhibitor activity of novel thiazolidinedione-oxadiazole derivative molecules and reported hydrogen bonding with Arg195, pi interactions with Trp59 and Tyr62, in addition to numerous alkylic and van der Waals interactions, with remarkable binding affinities (43). In this study, Chlorogenic acid, Catechin hydrate, Caffeic acid, Naringin, and Quercetin were evaluated to gain insight into their interactions with α-amylase. The references cited above show that the same residue of the target molecule was used to interact with all the molecules. Five of the molecules had H-bonds. Chlorogenic acid, Caffeic acid, and Naringin interacted with five H-bonds, while Catechin hydrate had only one H-bond with Asp297. The molecules also exhibited many alkylic and van der Waals interactions, in addition to pi interactions, which were recorded for Catechin hydrate and Caffeic acid. The best binding constant was recorded for Naringin as −7.40 kcal/mol, while Caffeic acid had the smallest binding value as −6.24 kcal/mol. Chlorogenic acid, Catechin hydrate, and Quercetin had −7.24, −6.39, and −7.22 kcal/mol binding values, respectively. All the interaction details were presented in Supplementary Table S1, Supplementary Figures S1, S3–S7.

Shulgau et al. analyzed thiourea derivative molecules for α-glucosidase inhibition activity *in vitro* methods in addition to molecular docking methods, in which −7.5 kcal/mol binding affinity was determined with two H-bonds with Asp282 and Arg600, and pi-interaction with Trp376 (44). Also, Abdullah et al. studied montelukast for its potent anti-enzymatic activity with the molecular docking method and recorded two H-bonds with Phe525 and Arg281 with remarkably good −8.82 kcal/mol binding affinity (45). In another recent study, Abdallah et al. analyzed the essential oil of *Centaurea alexanderina* α-glucosidase inhibitor activity as a part of anti-hyperglycemic activities by the molecular docking method. They recorded different types of interactions with the residues Trp376, Trp481, Phe525, and Phe649 for four identified metabolites (46).

In this study, Chlorogenic acid, Catechin hydrate, Caffeic acid, Naringin, and Quercetin were evaluated to gain insight into their interactions with α-glucosidase. The references cited above show that the same residue of the target molecule interacted with all the molecules. Five of the molecules had H-bonds. Quercetin interacted with five H-bonds, while Caffeic acid had two H-bonds with Leu636, and Gln633. The molecules also exhibited many alkylic and van der Waals interactions, in addition to pi interactions, which were recorded for Chlorogenic acid and Quercetin. The best binding constant was recorded for Chlorogenic acid at −7.17 kcal/mol, while Naringin had the smallest binding constant at −6.30 kcal/mol. Catechin hydrate, Caffeic acid and Naringin had −6.83, −6.46, and −6.30 kcal/mol binding value, respectively. All the interaction details were presented in Supplementary Table S1, Supplementary Figures S2, S8–S12. According to molecular docking results, phenolic compounds were found to form strong interactions with α-amylase and α-glucosidase enzymes, a finding consistent with the literature. Kifle et al. (47) also reported that plant phenolics exhibit high binding affinity for the same enzymes. This parallel finding supports the mechanism by which phenolic enrichment resulting from the USOH process enhances antidiabetic activity.

To validate the molecular docking protocol, the co-crystallized ligands present in the target protein structures were initially removed, energy-minimized, and subsequently re-docked into their respective binding sites without altering any functional parameters. This validation procedure was performed to assess the reliability of the docking approach by ensuring the accurate reproduction of ligand binding within the active site and minimizing deviations from the experimentally observed native conformations. The docking procedure was regarded as valid when the RMSD score was ≤2.0 Å for all targeted molecules. The molecular docking results demonstrated that the phenolic compounds established strong and stable interactions with both α amylase and α glucosidase, which is consistent with findings reported in the literature. Patil et al. (48) similarly showed that the major phenolics present in *Acalypha indica* exhibited meaningful binding affinities toward the same enzymatic targets, with quercetin and kaempferol displaying particularly high inhibitory potential. This parallel supports the mechanistic explanation that the phenolic enrichment achieved through USOH processing contributes to the enhanced antidiabetic activity observed in the present study (48).

## 4 Antidiabetic activity

**Figure 13:**
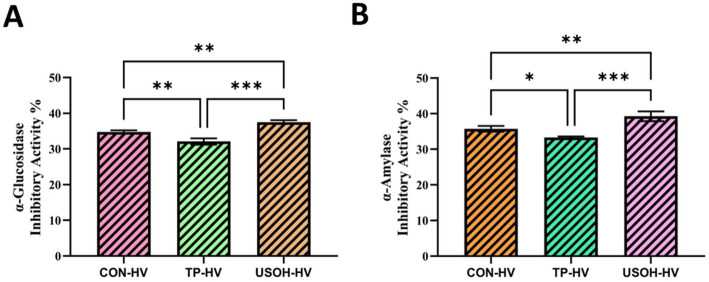
*In vitro* α-glucosidase **(A)** and α-amylase **(B)** inhibition activities of hawthorn vinegar samples. CON-HV, control hawthorn vinegar (untreated); USOH-HV, ultrasound–ohmic treated hawthorn vinegar, TP-HV, thermally pasteurized hawthorn vinegar. Values are presented as mean ± standard deviation (*n* = 3). Statistical significance is indicated as ns, not significant; ^*^p < 0.05; ^**^p < 0.01; ^***^p < 0.001.

The findings on the *in vitro* enzyme-inhibitory capacities of hawthorn vinegar samples clearly demonstrate that the applied processing technologies play a decisive role in the product’s biofunctional profile. Examining the experimental data and the α-glucosidase inhibition results presented in Figure 13A, the highest inhibitory effect was observed in samples treated with the ultrasound-ohmic (USOH-HV) combination (37.54%), proving the superiority of this innovative technology in improving functional properties (49). In contrast, conventional thermal pasteurization (TP-HV) statistically significantly reduced the inhibitory capacity on both enzyme systems compared to both the control (CON-HV) and combined treatment groups (Figures 13A,B), reflecting the destructive effect of heat treatment on bioactive fractions (*p* < 0.05). This loss of capacity observed during thermal pasteurization can be explained by the thermal degradation of thermolabile phenolic compounds, which are present in the fruit matrix and responsible for enzyme inhibition, due to high-temperature exposure (49, 50). It is known that the levels of bioactive components in fruit vinegars are determined by the raw materials used, microbial fermentation, and the processing methods applied (50). In this context, heat treatment is considered to promote the polymerization of phenolic structures, thereby reducing their ability to interact with enzymes and decreasing the therapeutic value of the product (51, 52).

The significant increase in enzyme inhibition observed with the combined ultrasound-ohmic treatment compared to the control group (Figure 13) is directly attributable to cavitation and improved mass transfer from a mechanistic perspective. The high inhibition values observed in the USOH-HV sample are associated with increased accessibility of bioactive components to the solvent due to the cavitation effect and more intensive extraction of intracellular fractions (49). Similarly, the enhancing effect of ultrasound assisted extraction and design based optimization on antidiabetic activity has also been demonstrated in recent studies. Patil et al. (48) reported that applying a Box–Behnken design to optimize ultrasound assisted extraction of *Acalypha indica* markedly increased the yield of phenolic compounds, which in turn strengthened the inhibitory effects on α amylase and α glucosidase. These findings further support the strong relationship between optimized processing conditions, phenolic enrichment, and improved antidiabetic potential (48). A similar trend has been observed in studies on watermelon vinegar; ultrasound application has been reported to enhance antidiabetic potential through cavitation reactions, and enzyme inhibition capacity can be maximized by optimizing treatment parameters (51). This demonstrates that ultrasonic treatment not only preserves existing components but also modifies the matrix effect, creating a stronger functional profile.

The antidiabetic potential of hawthorn vinegar is directly related to the phenolic compounds it predominantly contains, such as chlorogenic acid, caffeic acid, and quercetin. In particular, components such as quercetin and hyperoside have been reported to play a critical role in delaying carbohydrate digestion by binding to the active site of the α-glucosidase enzyme (49). Indeed, studies on various plant-derived vinegar extracts confirm that flavonoids such as quercetin and kaempferol inhibit α-amylase activity by forming strong molecular interactions with the enzyme (53). It can be said that the USOH-HV process provides more effective binding to enzymes by preserving and increasing the extractability of these specific phenolics. Furthermore, considering the fact that polysaccharides in hawthorn fruit also have significant inhibitory potential on α-amylase (Figure 13B), it is thought that combined processing technologies provide functional contributions by modifying the structural properties of these macromolecules (49).

Functional evaluation of control, thermally and combinedly processed samples reveals that USOH-HV provides not only a statistically significant difference but also a qualitative improvement in the product’s positive effects on health. While the functional weakening caused by thermal pasteurization reduces the product to the level of a standard foodstuff, the high inhibitory capacity provided by ultrasound-ohmic treatment makes hawthorn vinegar a powerful functional component that can aid in the management of chronic diseases. It is hypothesized that this antidiabetic mechanism of action may also be supported by modulation of transporter systems that affect intestinal glucose absorption and by stimulation of incretin hormones that trigger insulin release (54). It has also been determined that fruit ripeness and storage conditions have critical effects on enzyme inhibition capacity, and that vinegars obtained from unripe fruits, in particular, offer a higher phenolic content and stronger inhibition potential (52). In conclusion, the data obtained show that innovative technologies offer a superior alternative to traditional methods for maximizing the antidiabetic and biofunctional properties of hawthorn vinegar; this approach enhances the product’s bioavailability and strengthens its potential for developing value-added functional products in the food industry (49, 51).

### 4.1 Individual phenolic compound profile of hawthorn vinegar samples

When the individual phenolic compound profiles of hawthorn vinegar samples were evaluated in Table 5, it was determined that the different treatments applied caused significant changes in the phenolic composition. The results showed that the sample treated with ultrasound-ohmic treatment (USOH-HV) exhibited higher phenolic content than the control (CON-HV) and thermal pasteurization (TP-HV) samples. In particular, the higher concentrations of hydroxycinnamic acid, vanillin, chlorogenic acid, p-coumaric acid, trans-ferulic acid, catechin hydrate, caffeic acid, rutin, naringin, salicylic acid, resveratrol, and quercetin detected in the USOH-HV sample indicate that this method may contribute to the preservation and release of phenolic compounds from the matrix. The catechin hydrate content was 12.51 and 13.63 μg/mL in the control and thermally treated samples, respectively, and 28.07 μg/mL in the USOH-HV sample. Similarly, the caffeic acid level was measured as 2.01 μg/mL in the CON-HV sample, 3.75 μg/mL in the TP-HV sample, and 6.74 μg/mL in the USOH-HV sample. These findings suggest that ultrasound-ohmic treatment can increase the extraction efficiency of phenolic compounds and be more effective in preserving biologically active components. Furthermore, a previous study on apple and cranberry juice systems reported that increasing the applied electric field strength significantly reduced the time to reach the target temperature; this could help preserve product quality by shortening processing time (55). This mechanistic framework is important in explaining the high levels of catechin hydrate, caffeic acid, naringin, resveratrol, and quercetin identified in the USOH-HV sample. A shorter and more uniform process profile may have limited the degradation of phenolics while also supporting their release from the matrix by the effect of ultrasound.

**Table 5:** Individual phenolic compound profiles of hawthorn vinegar subjected to different processing treatments.

Phenolic compounds (μg/mL)	CON-HV	TP-HV	USOH-HV
Vanillin	0.25 ± 0.01ᵃ	0.23 ± 0.01ᵃ	1.05 ± 0.04ᵇ
Catechin hydrate	12.51 ± 0.84ᵃ	13.63 ± 0.74ᵃ	28.07 ± 1.02ᵇ
Resveratrol	0.13 ± 0.01ᵇ	n.d.	0.58 ± 0.02ᶜ
Caffeic acid	2.01 ± 0.13ᵃ	3.75 ± 0.21ᵇ	6.74 ± 0.25ᶜ
Chlorogenic acid	4.09 ± 0.28ᵇ	0.97 ± 0.05ᵃ	4.55 ± 0.16ᵇ
Naringin	2.23 ± 0.15ᵇ	1.60 ± 0.08ᵃ	4.75 ± 0.17ᶜ
Hydroxycinnamic acid	0.83 ± 0.06ᵃ	0.67 ± 0.04ᵃ	1.85 ± 0.06ᵇ
Resveratrol	0.13 ± 0.01ᵇ	n.d.	0.58 ± 0.02ᶜ
Quercetin	2.89 ± 0.20ᵇ	n.d.	6.40 ± 0.23ᶜ
Rutin	1.23 ± 0.08ᵇ	0.39 ± 0.02ᵃ	5.13 ± 0.18ᶜ
Rosmarinic acid	1.21 ± 0.08ᵇ	0.52 ± 0.03ᵃ	1.39 ± 0.05ᵇ
Salicylic acid	0.14 ± 0.01ᵃ	0.25 ± 0.01ᵃ	2.91 ± 0.11ᵇ
Trans-ferulic acid	1.35 ± 0.09ᵃ	1.13 ± 0.06ᵃ	3.11 ± 0.11ᵇ
p-Coumaric acid	0.96 ± 0.06ᵃ	0.98 ± 0.06ᵃ	3.72 ± 0.13ᵇ
Salicylic acid	0.14 ± 0.01ᵃ	0.25 ± 0.01ᵃ	2.91 ± 0.11ᵇ

Different lowercase superscript letters within the same row indicate statistically significant differences among treatments (*p* < 0.05). TP-HV, thermally pasteurized hawthorn vinegar; CON-HV, control hawthorn vinegar (untreated); USOH-HV, ultrasound–ohmic treated hawthorn vinegar. n.d., not detected (below the detection/quantification limit of the method).

The observed increasing trend for rutin and quercetin also supports this finding; in particular, the reappearance of some compounds at significant levels in the USOH-HV group, which were undetectable in the TP-HV sample, indicates that ultrasound-ohmic treatment offers a more advantageous process approach than classical thermal treatment. These findings suggest that the synergistic effect of ultrasound and ohmic heating facilitates the separation of phenolic compounds from the matrix. It is known that ultrasound application weakens cell wall structures through cavitation, while ohmic heating helps reach the target temperature more quickly by providing rapid, volumetric heating within the product. In a study on mandarin juice, ohmic heating was reported to provide rapid, homogeneous internal heating, shorten processing time, and offer a significant advantage in preserving the product’s nutritional and structural properties (56). Similarly, in the model developed for passion fruit juice, it was shown that increasing the temperature increased electrical conductivity, which in turn increased ion mobility, resulting in more efficient ohmic processing performance (57). Therefore, the phenolic enrichment observed in the USOH-HV sample in this study is due not only to thermal effects but also to protective and liberating mechanisms arising from the combination of rapid internal heating and ultrasound-induced enhanced mass transfer.

The reduced or undetectable presence of certain phenolic compounds in the thermal pasteurization sample suggests that conventional heat treatment may be more detrimental to phenolic stability. For example, chlorogenic acid decreased to 0.97 ug/mL in the TP-HV sample, whereas it was measured at 4.09 and 4.55 ug/mL in the control and USOH-HV samples, respectively. Similarly, rutin decreased to 0.39 ug/mL in the TP-HV sample, while it reached 5.13 ug/mL in the USOH-HV sample. The undetectable presence of resveratrol and quercetin in the TP-HV sample also supports this trend. In a study on apple juice, ohmic heating was reported to result in greater reductions in total phenolic compounds, total flavonoids, and ascorbic acid in clear apple juice compared to conventional methods, according to the first-order kinetic model (58). This finding demonstrates that the ohmic process, as a gentler heat treatment, can limit quality losses.

Butt et al. (59) stated that sono-ohmic pasteurization applied to milk reduced the microbial load while increasing total phenolic content, produced controlled changes in physicochemical and sensory properties, and was generally effective (59). Although the study in question was conducted on a different food matrix, it supports the current results by demonstrating that the sono-ohmic approach can provide process efficiency while preserving product quality. Therefore, the positive effect of sono-ohmic treatment on phenolic compounds in hawthorn vinegar can be considered not only as a result specific to matrix properties but also as a reflection of the overall quality-preserving potential of this technology.

Functional beverages and vinegar processed using optimized ultrasonic-ohmic (USOH) treatment offer significant potential for industrial-scale operations. In addition to shortening thermal processing times compared to traditional thermal pasteurization, USOH’s integration with machine learning models (such as Lasso Poly2) is crucial for maintaining product quality and compatibility with automated industrial control systems. In this study, the accuracy, precision, and repeatability of the HPLC–DAD method used for the quantification of phenolic compounds were found to be consistent with the high analytical reliability reported for analytical methods developed using the Quality by Design (QbD) framework. In a related study, Umarani et al. (60) optimized an eco-friendly and stability-indicating ultraviolet (UV) spectroscopic method for trigonelline analysis based on a Design of Experiments (DoE) approach, demonstrating that the method achieved high analytical performance through its excellent accuracy (recovery 99.56–101.85%), low limits of detection and quantification (LOD/LOQ), and strong robustness, thereby reinforcing the importance of QbD-driven analytical quality assurance (60).

## 5 Conclusion

This study demonstrated that integrating ultrasound–ohmic (USOH) processing with machine-learning-based optimization provides an effective strategy to enhance the antidiabetic and functional properties of hawthorn vinegar. Among the 13 machine learning algorithms evaluated, the Lasso Poly2 model exhibited the highest predictive performance for both α-glucosidase and α-amylase inhibitory activities, achieving high cross-validation accuracy and low prediction errors. Moreover, Differential Evolution, Particle Swarm Optimization, and Gray Wolf Optimization converged to nearly identical optimum conditions, confirming the robustness and reliability of the developed optimization framework. Under the optimized conditions, USOH treatment significantly improved the phenolic profile of hawthorn vinegar compared with untreated and thermally pasteurized samples, resulting in increased concentrations of major phenolic compounds and enhanced inhibitory activities against both target enzymes. Molecular docking analysis further supported the experimental findings by demonstrating favorable interactions between the identified phenolics and α-glucosidase and α-amylase, with chlorogenic acid and naringin exhibiting the strongest binding affinities toward their respective enzymes. Collectively, these findings highlight the potential of combining non-thermal processing technologies with machine learning-driven optimization to develop value-added functional vinegar products with enhanced bioactive composition and antidiabetic potential. From an industrial perspective, the scalability, energy efficiency, and reduced thermal impact of ultrasound–ohmic processing, together with the availability of both technologies at pilot and industrial scales, underscore its promise for commercial functional vinegar production; however, further studies on pilot-scale validation, economic feasibility, shelf-life stability, consumer acceptance, and regulatory compliance are required to facilitate successful industrial implementation.

## Data Availability

The raw data supporting the conclusions of this article will be made available by the authors, without undue reservation.

## Footnotes

1
https://www.rcsb.org/
