# Supplementary material for: Machine learning-assisted optimization of ultrasound–ohmic processing of hawthorn vinegar: antidiabetic activity, phenolic profiling, and molecular docking insights
Source: Front Nutr. 2026 Jul 8;13:1889843. doi: 10.3389/fnut.2026.1889843 (PMC13389359; doi:10.3389/fnut.2026.1889843)
Supplement: Supplementary file 1 [file Table_1.DOCX]

**Supplementary Files**

**Table S1.** Interaction details, and binding values of the selected phenolic compounds against α-amylase, and α-glucosidase

| Comp. | Bind. Aff.* | Amino Acids Residue | |
| --- | --- | --- | --- |
| α-amylase | | |  |
| Chlorogenic acid | -7.24 | His15, Gln41, Glu233, Arg337 (H-bonds), Tyr231, Thr254, Phe256, Phe295, Asp297, Asn98, Met339 (alkylic interactions), Tyr62, Gln232, Ile235, Leu293, Val296, His299, Met328, Val338 (van der Waals interactions) | |
| Catechin hydrate | -6.39 | Asp297 (H-bond), His15, Met328 (pi-interactions), Gln41, Phe295, Val296, Asn298, Arg337, Val338, Met339 (alkylic interactions), Ile13, Val14, Arg195, His299, Asp300, Tyr321, Val325 (van der Waals interactions) | |
| Caffeic acid | -6.24 | Val296, Asp297, His299, Tyr321 (H-bonds), Arg337, Met339 (pi-interactions), His15, Gln41, Val325, Met328, Val338 (van der Waals interactions) | |
| Naringin | -7.40 | Arg195, Gln302, Tyr321, Val338 (H-bonds), His15, Gln41, Ser43, Pro44, Tyr62, Asp96, Phe295, Asp297, Asn298, His299, Met328, Thr336, Arg337, Met339 (alkylic interactions), Ser12, Phe17, Glu18, Arg61, Val42, Phe256, Val296, Val325, Leu329 (van der Waals interactions) | |
| Quercetin | -7.22 | Tyr321, Val325 (H-bond), Val296, Asp297, Asn298, Met328, Leu329 Arg337, Val338, Met339 (Alkylic interactions), Ser12, His15, Gln41, Phe295, His299, Thr336, Ile391 (van der Waals interactions) | |
| α-glucosidase | | |  |
| Chlorogenic acid | -7.17 | Thr258, Val351, Leu637 (H-bonds), Val358 (pi-interactions), Phe297, Tyr354, Leu355, Arg608, Leu636, Gly638 (alkylic interactions), Gly259, Tyr298, Leu299, Gly309, Val350, Gly359, Tyr360, Pro361, Ala604, Gly605, His606, Gly607, Gln633 (van der Waals interactions) | |
| Catechin hydrate | -6.83 | His295, Tyr354, Gly607 (H-bonds), Leu355, Val358, Gly359, Pro361, Thr602, His606, Phe634, Leu637, Gly638, Val639 (alkylic interactions), Tyr360, Phe603, Ala604, Gly605, Arg608, His612 (van der Waals interactions) | |
| Caffeic acid | -6.46 | Leu636, Gln633 (H-bond), Leu299, Val350, Val351, Leu637 (alkylic interactions), Thr258, Gly259, Phe297, Tyr354, Leu355, Gly638 (van der Waals interactions) | |
| Naringin | -6.30 | Phe603, Gly611, Leu641, Gly643 (H-bonds), Ala261, His295, Tyr354, Leu355, Val358, Gly359, Ile631, Thr602, Gly605, His606, His612, Phe634, Leu637, Val639, Val642 (alkylic interactions), Phe297, Tyr360, Leu577, Ser599, Ser601, Ala604, Gly607, Arg608, Trp613, Thr614, Glu630, Phe670 (van der Waals interactions) | |
| Quercetin | -6.78 | Thr258, His295, Val351, Gly605, Gln633 (H-bonds), Phe297 (pi-interaction), Leu299, Tyr354, Leu355, Val358, Leu636, Leu637 (alkylic interaction), Gly259, Leu267, Val350, Phe603, Ala604, His606, Gly607, Gly638, Val639 (van der Waals interactions) | |

* Binding Affinity in kcal/mol.


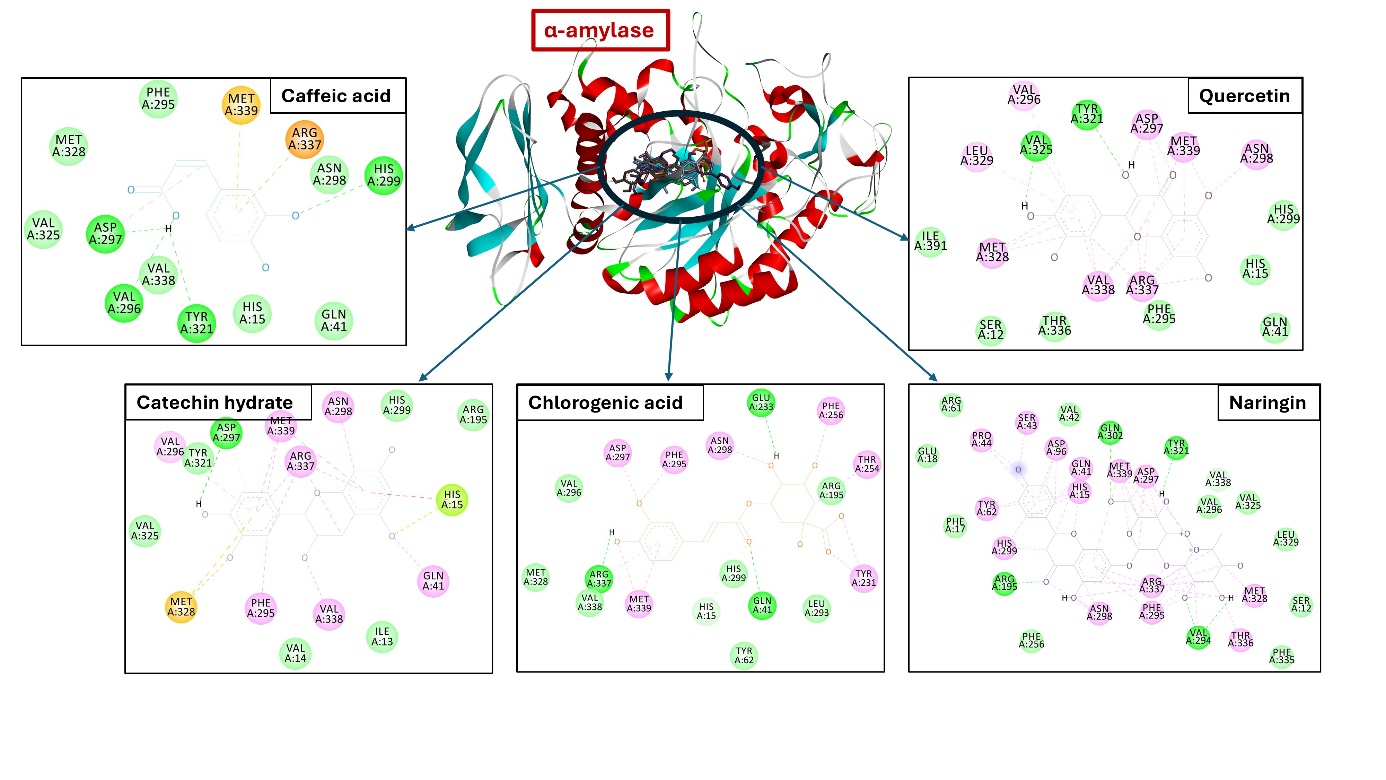


**Figure S1.** Interaction details of the selected phenolic compounds against α-amylase


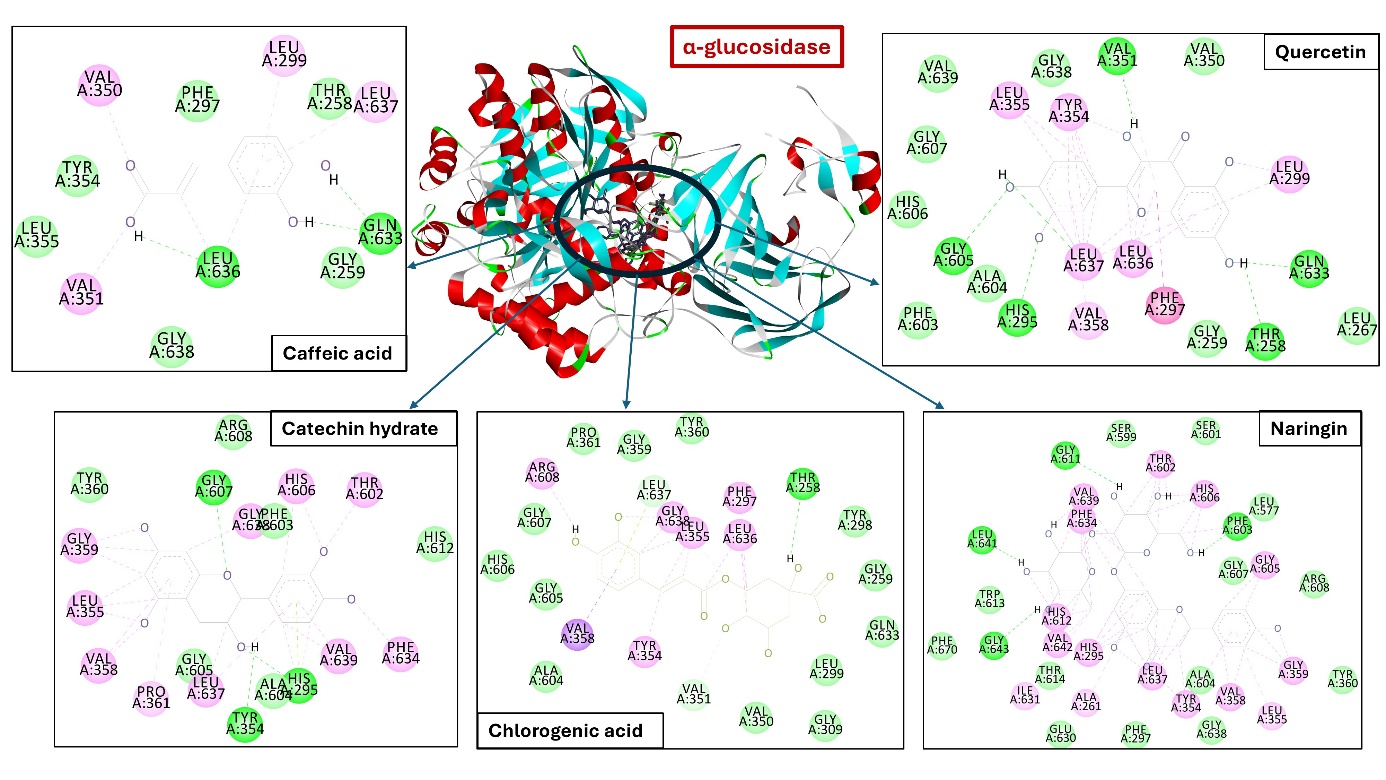


**Figure S2.** Interaction details of the selected phenolic compounds against α-glucosidase

**
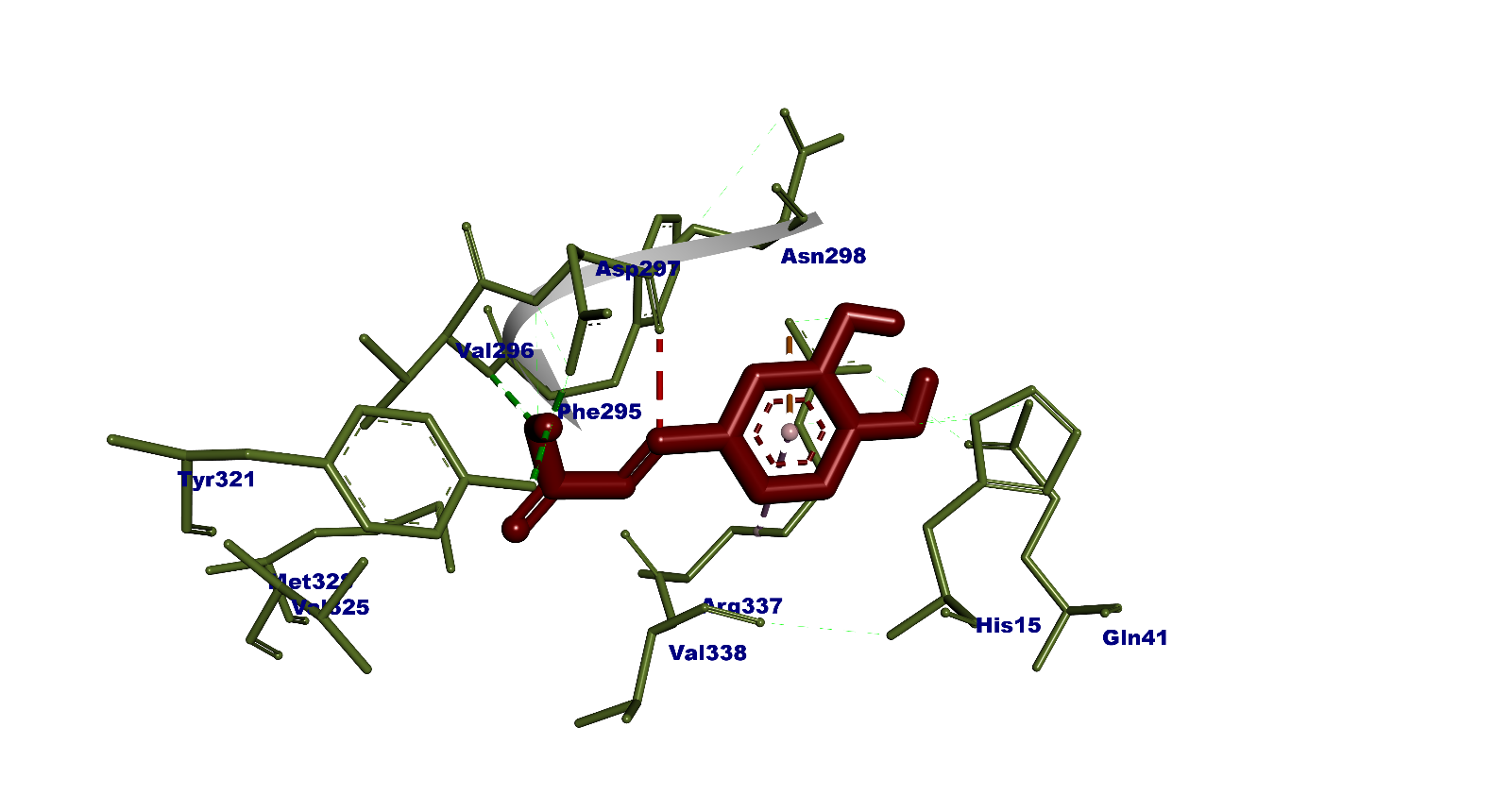
**

**Figure S3.** Interaction details of Caffeic acid against α-amylase


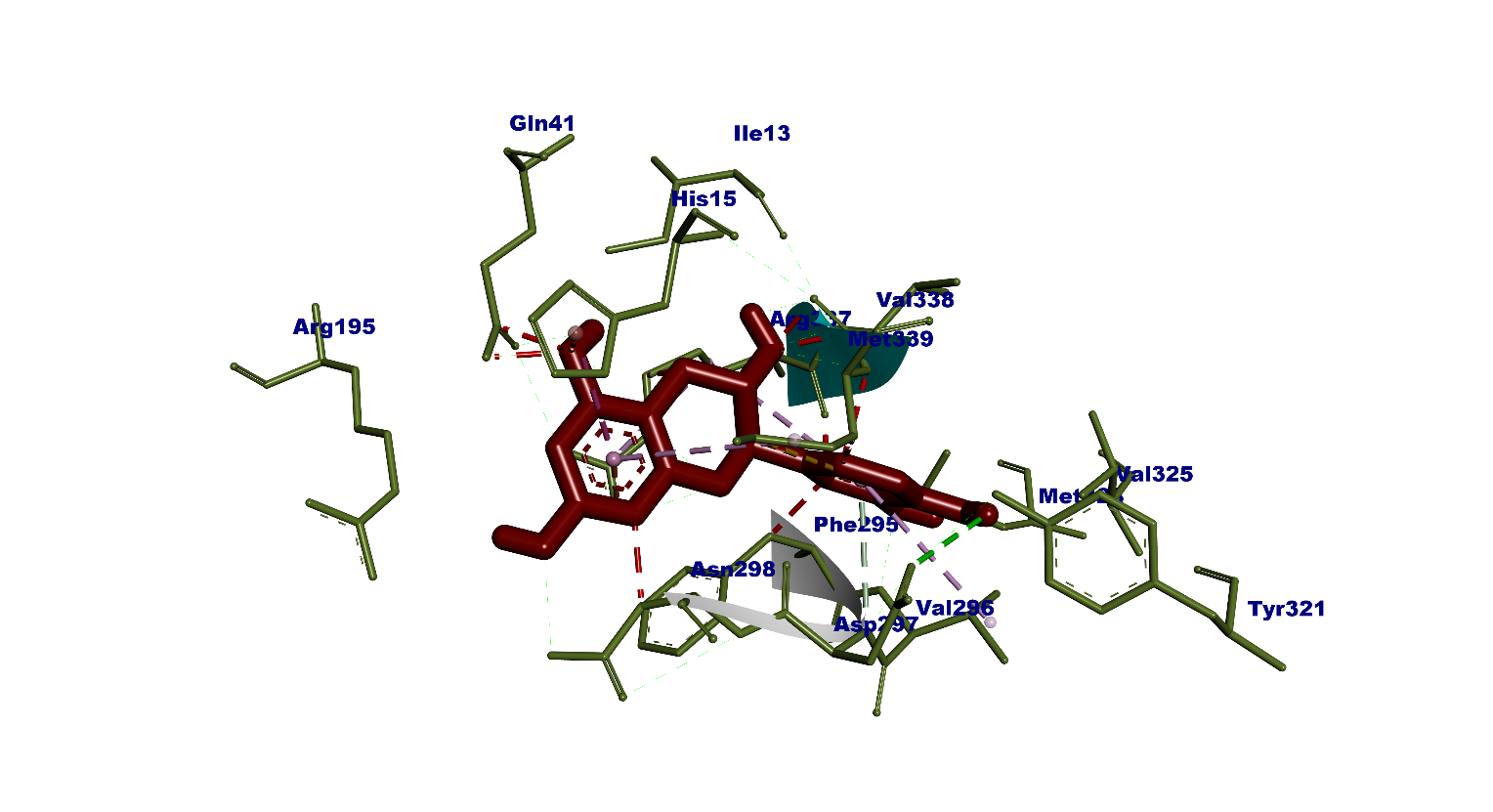


**Figure S4.** Interaction details of Catechin hydrate against α-amylase


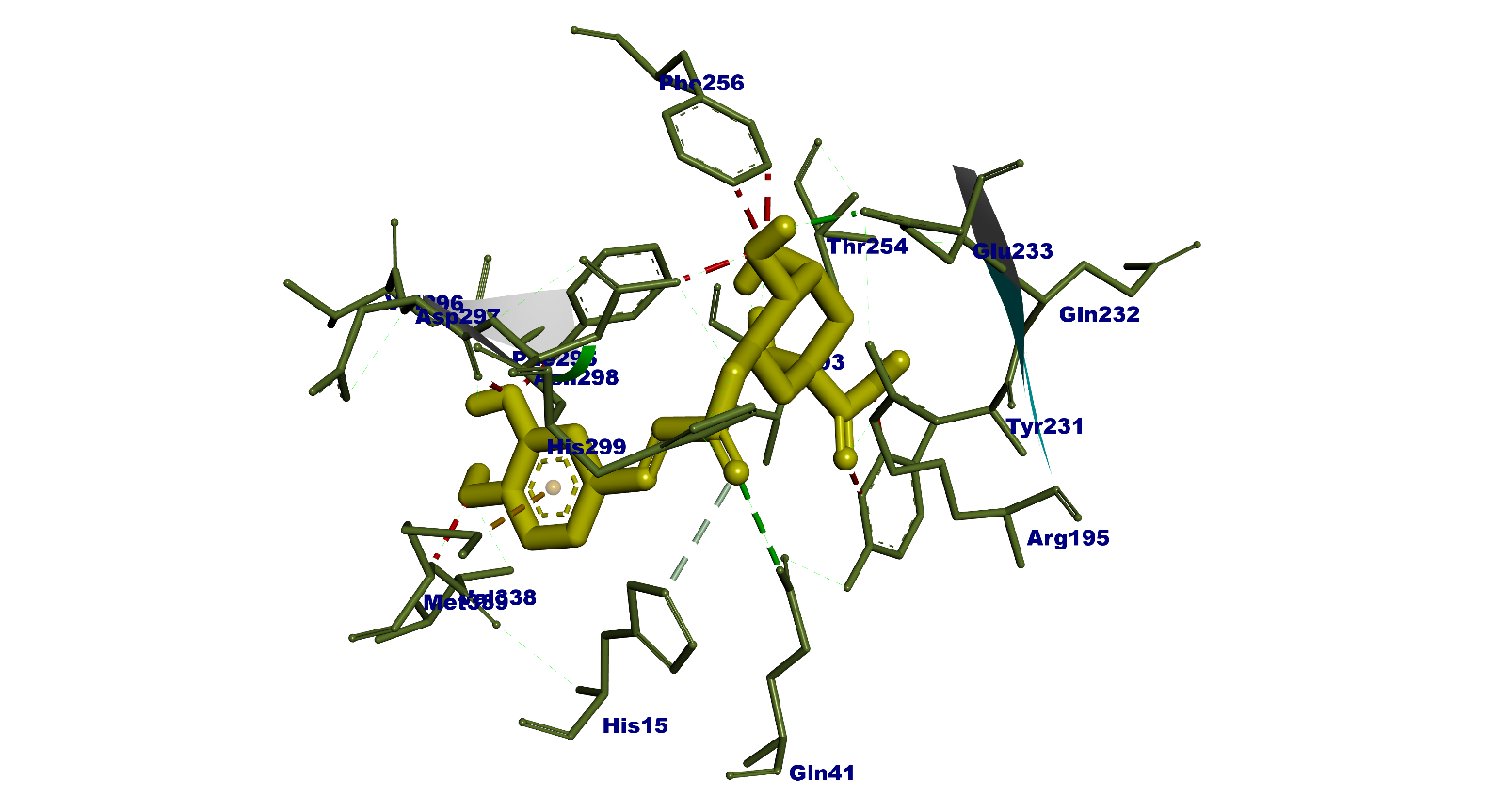


**Figure S5.** Interaction details of Chlorogenic acid against α-amylase


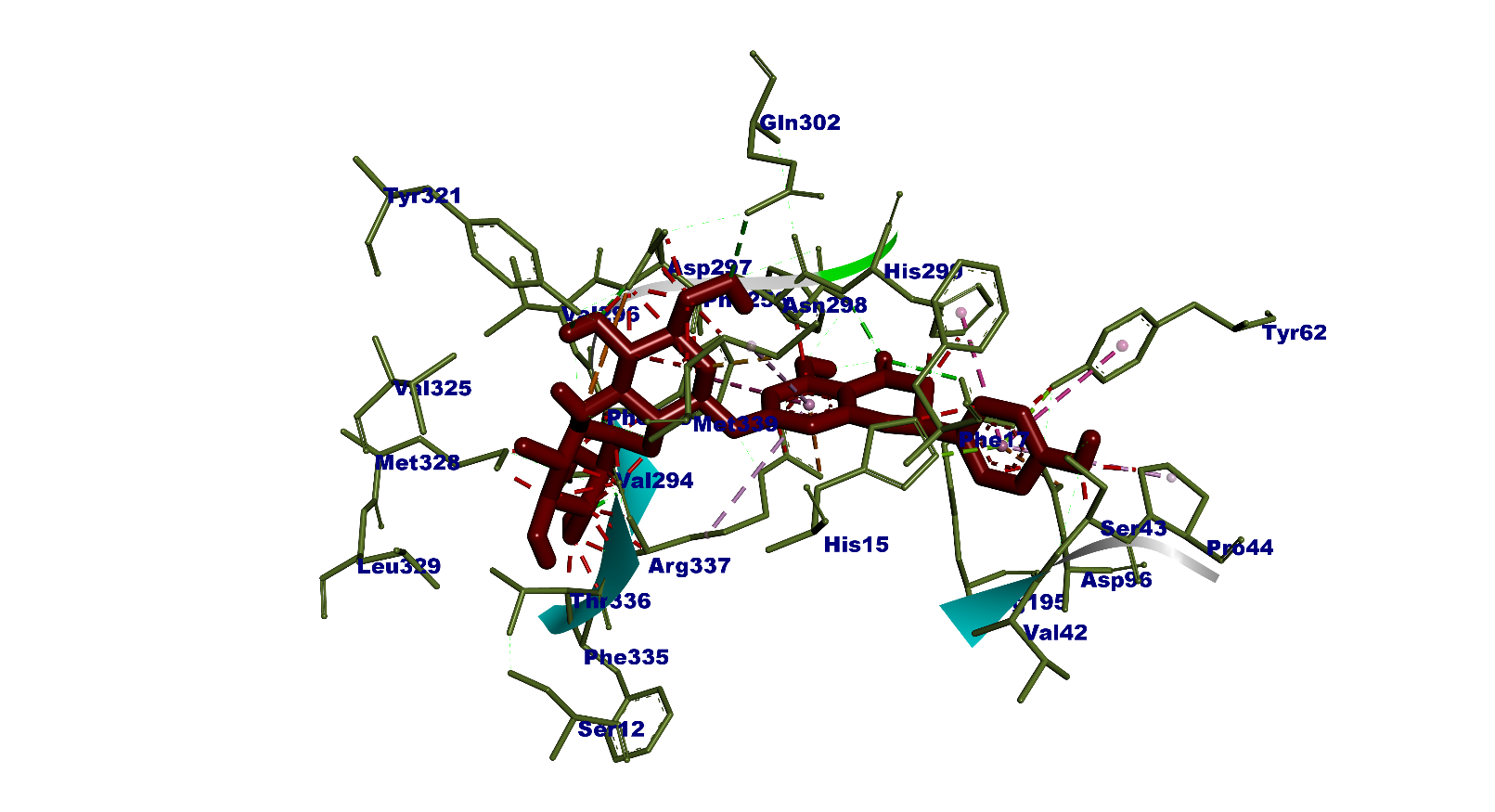


**Figure S6.** Interaction details of Naringin against α-amylase


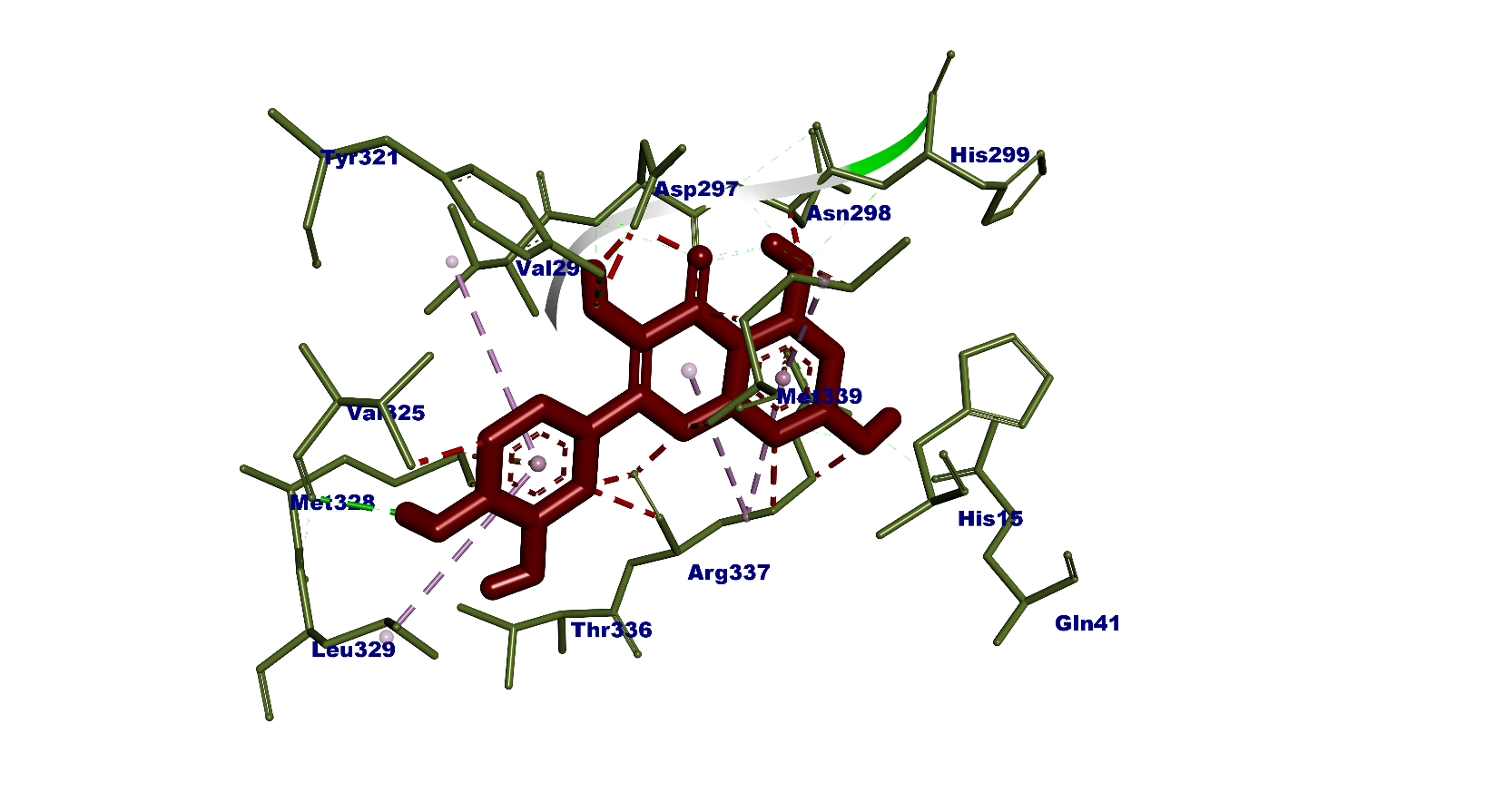


**Figure S7.** Interaction details of Quercetin against α-amylase


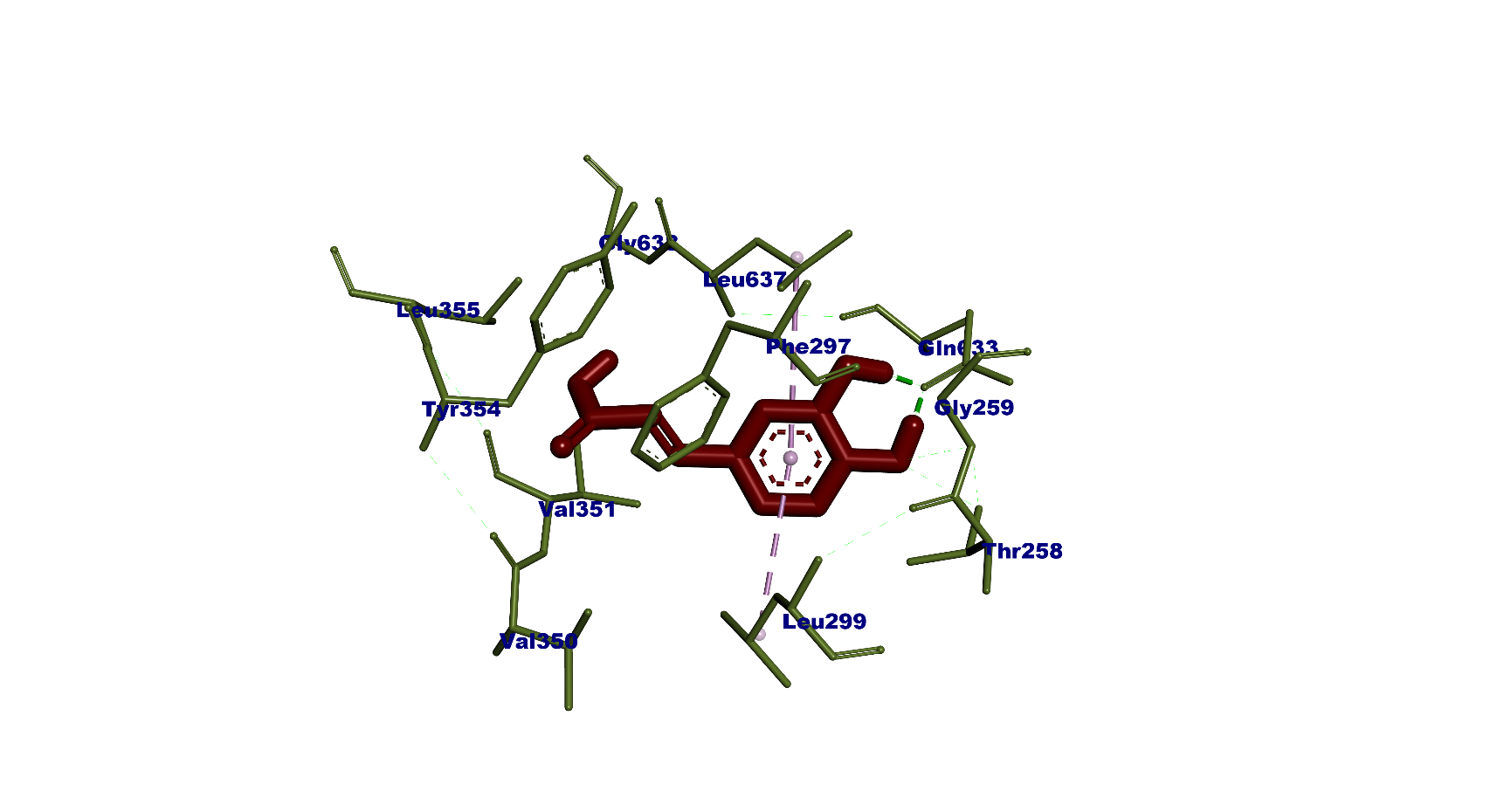


**Figure S8.** Interaction details of Caffeic acid against α-glucosidase


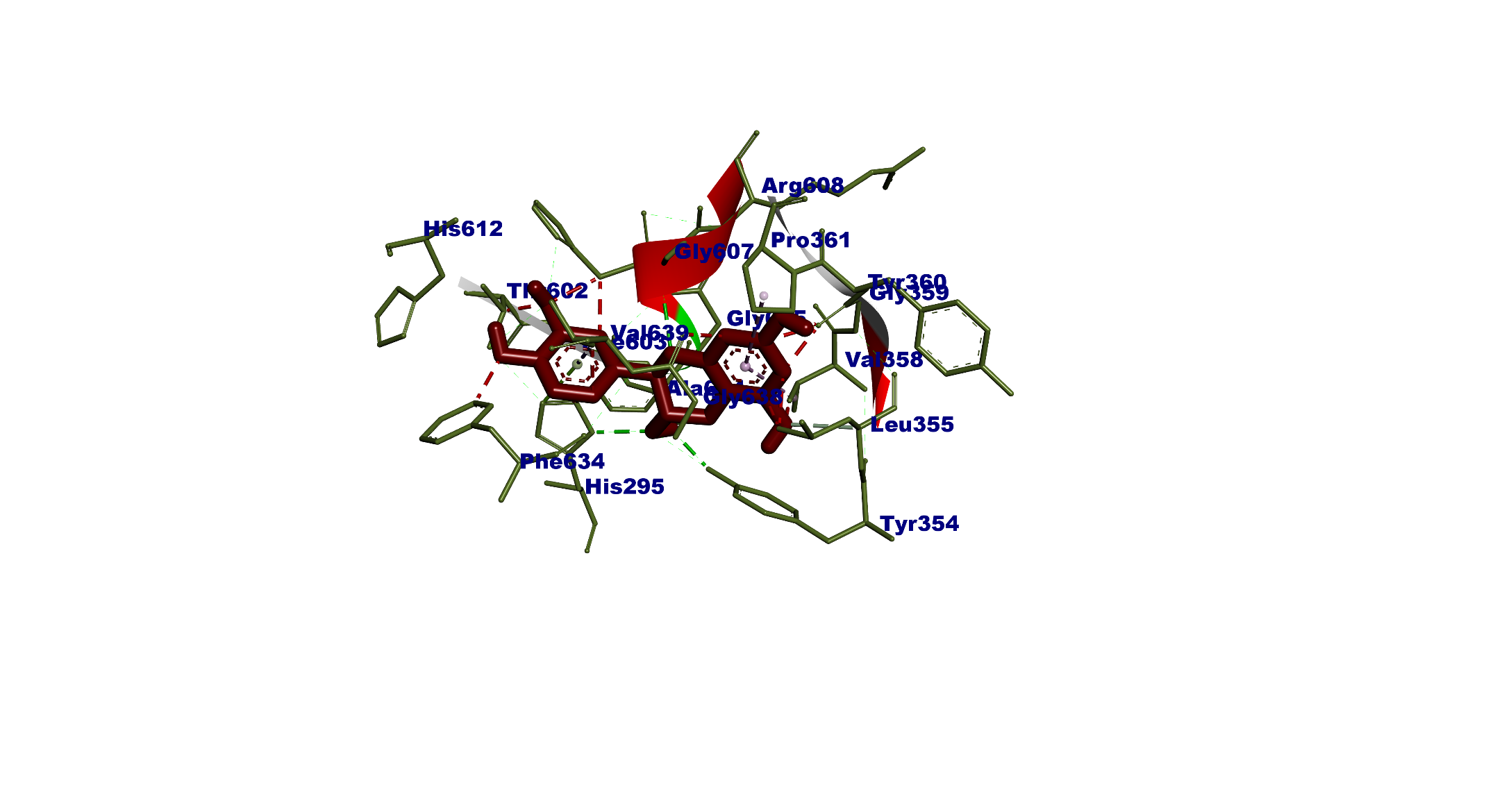


**Figure S9.** Interaction details of Catechin hydrate against α-glucosidase


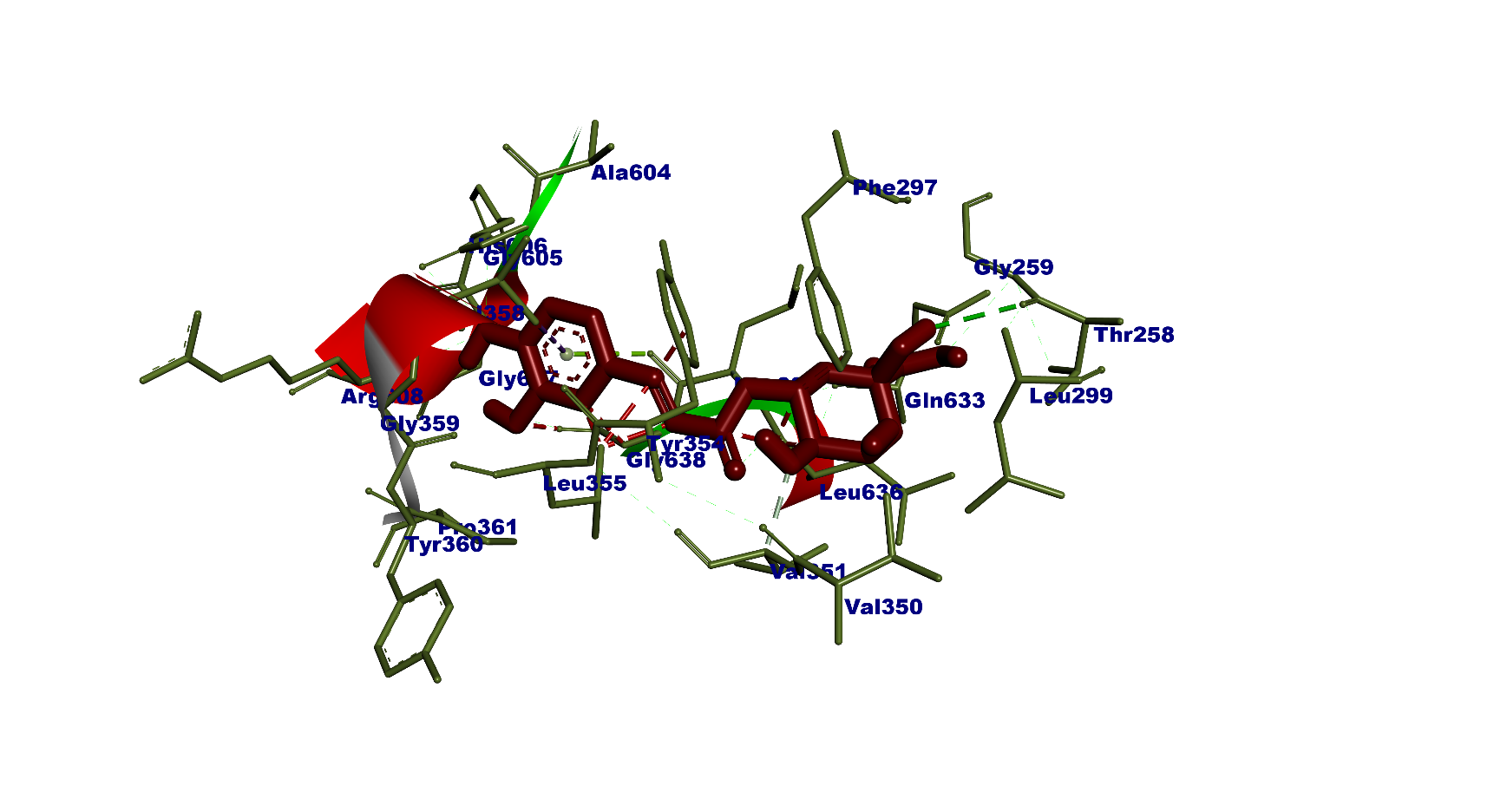


**Figure S10.** Interaction details of Chlorogenic acid against α-glucosidase


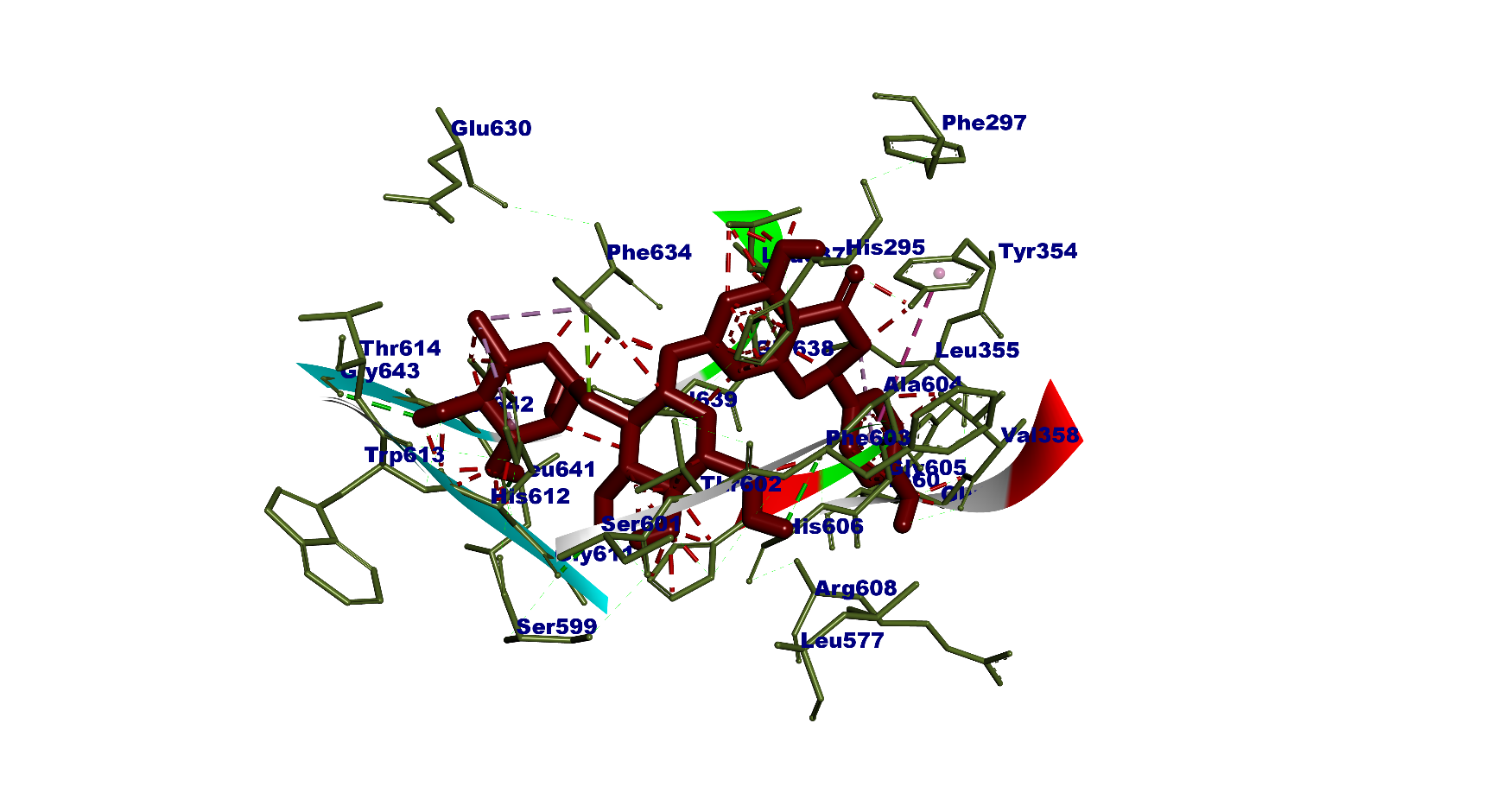


**Figure S11.** Interaction details of Naringin against α-glucosidase


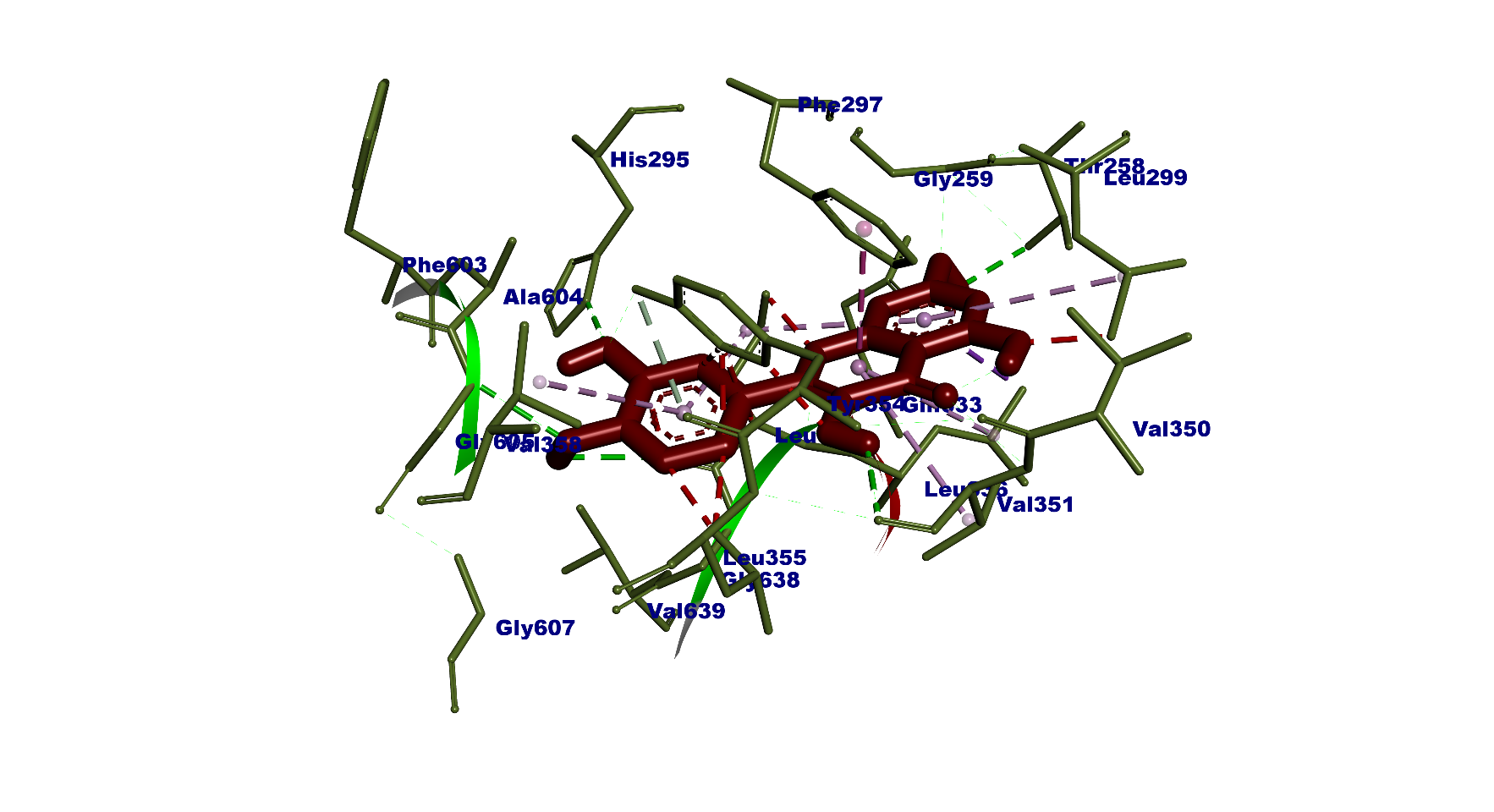


**Figure S12.** Interaction details of Quercetin against α-glucosidase
